# The mRNA degradation factor Xrn1 regulates transcription elongation in parallel to Ccr4

**DOI:** 10.1093/nar/gkz660

**Published:** 2019-08-08

**Authors:** Victoria Begley, Daniel Corzo, Antonio Jordán-Pla, Abel Cuevas-Bermúdez, Lola de Miguel-Jiménez, David Pérez-Aguado, Mercedes Machuca-Ostos, Francisco Navarro, María José Chávez, José E Pérez-Ortín, Sebastián Chávez

**Affiliations:** 1 Instituto de Biomedicina de Sevilla, Universidad de Sevilla-CSIC-Hospital Universitario V. del Rocío, Seville 41012, Spain; 2 Escuela Técnica Superior de Informática, Universidad de Sevilla, Seville 41012, Spain; 3 E.R.I. Biotecmed, Universitat de València; Burjassot, Valencia 46100, Spain; 4 Departamento de Biología Experimental, Facultad de Ciencias Experimentales, Universidad de Jaén, Jaén 23071, Spain; 5 Departamento de Matemática Aplicada I and Instituto de Matemáticas, Universidad de Sevilla, Seville 41012, Spain

## Abstract

Co-transcriptional imprinting of mRNA by Rpb4 and Rpb7 subunits of RNA polymerase II (RNAPII) and by the Ccr4–Not complex conditions its post-transcriptional fate. In turn, mRNA degradation factors like Xrn1 are able to influence RNAPII-dependent transcription, making a feedback loop that contributes to mRNA homeostasis. In this work, we have used repressible yeast *GAL* genes to perform accurate measurements of transcription and mRNA degradation in a set of mutants. This genetic analysis uncovered a link from mRNA decay to transcription elongation. We combined this experimental approach with computational multi-agent modelling and tested different possibilities of Xrn1 and Ccr4 action in gene transcription. This double strategy brought us to conclude that both Xrn1–decaysome and Ccr4–Not regulate RNAPII elongation, and that they do it in parallel. We validated this conclusion measuring TFIIS genome-wide recruitment to elongating RNAPII. We found that *xrn1Δ* and *ccr4Δ* exhibited very different patterns of TFIIS versus RNAPII occupancy, which confirmed their distinct role in controlling transcription elongation. We also found that the relative influence of Xrn1 and Ccr4 is different in the genes encoding ribosomal proteins as compared to the rest of the genome.

## INTRODUCTION

Gene expression was thought to be a linear process where each step came after the next without interfering in each other’s paths. However, abundant studies have made it evident that these pathways are interconnected and can influence one another (reviewed in (1–3)). The coupling of transcription and mRNA decay through transcription and degradation factors is what we call cross-talk. This mechanism is thought to be important because it allows cells to maintain mRNA levels within a certain limit despite various perturbations that could occur inside or outside the cell (4), and facilitates adaptation to new circumstances.

A few transcription factors have already been found to be implicated in this cross-talk since they affect the fate of nascent mRNA. This process has been named mRNA imprinting (5), and the most prominent factors involved are Rpb4/7, components of the RNA polymerase II (RNAPII) (6) and the multifunctional Ccr4–Not complex (7). These factors bind to the nascent mRNA in the nucleus, influencing its export into the cytoplasm, translation and degradation. Other factors have been linked to an opposite phenomenon: stimulation of gene transcription after cytoplasmic degradation of mRNA. The most distinguished factors that take part in this mechanism are Xrn1, the major 5′-3′ exonuclease part of the decaysome complex (8,9), and again, the Ccr4–Not complex, one of the major deadenylases (10). Both complexes are part of the mRNA pathways in the cytoplasm, yet have been found to shuttle into the nucleus and modulate transcription initiation and/or elongation (4,11). Xrn1 has been shown to control mRNA degradation and synthesis of an important subset of the yeast genome, acting as a synthegradase (12,13).

Interestingly, both of the main complexes involved in mRNA imprinting have been found to interact (14). In the nucleus, Rpb4/7 is necessary for the Ccr4–Not complex to promote transcription elongation by reactivating backtracked RNAPII (15). RNAPII backtracking occurs when the polymerase takes a few steps backwards, and the 3′ end of the growing RNA chain becomes disengaged from the catalytic site. In this event, the elongation complex becomes paused and must resolve the situation before resuming transcription. Transcription factor IIS (TFIIS), an RNA cleavage factor, can help resolve this situation by stimulating intrinsic hydrolysing activity of the polymerase, which cleaves the backtracked 3′ stretch of the nascent RNA, allowing transcription to restart from an upstream point (16). It has been proposed that Ccr4–Not can also prevent backtracking and/or rescue backtracked RNAPII. However, it is thought to do so in a different manner: by binding the polymerase and realigning the transcript into the active centre (11). It may also act in cooperation with TFIIS (17).

RNAPII arrest and backtracking have been suggested to be important for transcription regulation of genes (18,19). Furthermore, RNAPII backtracking was found to be important for the recently demonstrated role of Ccr4–Not in mRNA imprinting. The binding of the complex to nascent mRNA can influence translation and subsequent degradation of mRNAs (7). These findings made RNAPII backtracking an interesting subject in the context of cross-talk between transcription and mRNA decay.

Cross-talk of transcription, mRNA degradation and translation has been mathematically modelled using a stochastic kinetic approach (20). Their results favour the circular gene expression hypothesis over the traditional model of linear transcription and degradation, with Xrn1–decaysome in the centre of the feedback regulation. Agent-based simulation is an alternative way of modelling this mechanism. This kind of simulation can deal with complex systems made up of autonomous entities called agents (21). In computation, agents are independent and autonomous, with a capability to adapt and modify their behaviours. They can move freely and interact with other agents or the environment. The interaction of many agents in the same environment leads to emergent properties of the system, which were not previously defined. These emergent properties can provide information on the system dynamics and allow us to draw conclusions.

Accurate measurement of mRNA decay is experimentally difficult. It usually involves transcriptional shut-off by stressing procedures, which can generate experimental artefacts (22). However, mRNA decay of regulated genes can be measured after physiological repression of the gene’s promoter. In yeast cells growing in galactose-containing medium, the *GAL1* gene can be rapidly repressed by adding glucose, allowing easy calculation of its mRNA half-life. Although changing carbon sources may have some overall influence on mRNA stability (23), it seems not to affect mRNA half-lives of *GAL* genes. In fact, mRNA half-life measured after repression of a *GAL* gene driven by a TET-off promoter produced very similar values in comparison with the glucose shut-off method (24).

In this study, we first used a *GAL1-*based experimental approach to determine the transcriptional and degradation profiles of eight different mutants. These mutants were specifically chosen because of their known involvement in cross-talk and RNAPII backtracking. Then, we mathematically modelled this cross-talk using a multi-agent programming language, NetLogo (25).

Here we show the diverse effects in transcription and mRNA degradation caused by mutations in the transcription or degradation machineries. This extensive study then allowed us to find novel correlations linking RNAPII elongation to mRNA half-life, which supports a functional connection between transcription elongation and mRNA degradation. Our multi-agent model also supports the idea of a cross-talk between mRNA transcription elongation and decay, where feedback regulation is carried out by Ccr4 and Xrn1, which we identify as important elements of the mechanism. Both experimental and computational results indicate that the influence of Xrn1 in this feedback mechanism is not indirectly mediated by Ccr4 and that it may be affecting RNAPII backtracking. We conclude that Xrn1–decaysome and Ccr4–Not complexes play different roles in the regulation of RNAPII transcription elongation.

## MATERIALS AND METHODS

### Strains and growth conditions

*Saccharomyces cerevisiae* strains used in this study are listed in Supplementary Table S1. Strains were grown at 30°C, with liquid cultures growing in an orbital shaking incubator at 180 rpm in YP medium (1% yeast extract, 2% peptone) and containing either 2% galactose (YPGal) or 2% glucose (YPD) as a carbon source. All the *S. cerevisiae* strains utilized were in the BY4741 background.

For transcriptional shut-off assays, cells were grown in YPGal for at least seven generations until reaching an OD_600 nm_ of ∼0.5. A sample was taken, and immediately after, glucose 4% was added to the culture. Samples were taken at the indicated times after glucose addition.

### Determination of cell volume, number and growth

The median values of cell volumes were calculated by a Coulter-Counter Z series device (Beckman Coulter, USA). Cell number was determined by counting the number of cells using a Neubauer chamber. Growth curves were done for each mutant by measuring OD_600 nm_ every 2 h for a total of 5 time-points. The doubling time was estimated from this growth curve (see Supplementary Table S1 for data).

### RNA extraction and RT-qPCR

Cells were grown to log phase and 10 ml was harvested by centrifuging for 2 min at 4000 rpm and flash freezing the cells in liquid nitrogen. Total RNA was purified using hot phenol–chloroform method as described previously (26) and reverse-transcribed to cDNA using M-MLV Reverse Transcriptase (Promega). A relative real-time quantitative PCR was then carried out for all the genes studied (Supplementary Table S2) against *SCR1* using SYBR Green Premix Ex Taq (Takara) in a Light Cycler 480 II (Roche). Since *SCR1* is an abundant and stable RNA that has a constant concentration during cell growth (27), normalization of mRNA values to *SCR1* is an indirect way to calculate apparent mRNA concentration ([mRNA]).

To determine the mRNA half-lives, we used a transcription shut-off assay, collecting samples at 5, 10, 20, 30, 40, 50 and 60 min after glucose addition. mRNA levels were then determined with RT-qPCR and half-lives were estimated by calculating the time it takes for half of the initial amount of RNA to be degraded. In all mutants analysed in this work, the galactose/glucose signalling pathway was operative, as detected by measuring the disappearance of RNAPII from the 5′ end of *GAL1p-YLR454w* shortly after adding glucose (see Supplementary Figure S3A). Nevertheless, in those mutants where the signal did not immediately decrease, we calculated the half-lives from minute 5.

### Chromatin immunoprecipitation (ChIP)

Yeast strains were grown to exponential phase and 50 ml were taken for each sample. The ChIP experiments were performed as previously described (28), except that anti-Rpb3 (ab81859; Abcam), anti-HA (3F10 Roche) or anti-C-Myc (9E10 Santa Cruz Biotechnology) antibodies and Sheep anti-Mouse or anti-Rat IgG Dynabeads (Invitrogen) were used. Genes were analysed by quantitative real-time PCR in triplicate using primers listed in Supplementary Table S2.

To determine RNAPII speed, we performed a transcription shut-off assay by quickly cross-linking the cells at 2, 3 and 4 min after glucose addition. In two cases the times were 2, 3.5 and 5 min after stopping transcription. To obtain RNAPII speed values, we divided each % IP value by its previous time-point in order to take into account only the polymerases that remain transcribing at each moment. Using an engine at www.wolframalpha.com, we calculated the area of RNAPII remaining on the gene in order to determine how much RNAPII had left the gene at each time point. To obtain the speed for each point, we divided the resulting RNAPII amount by the time elapsed. Finally, for the general RNAPII speed for each mutant, we determined the weighted average of the RNAPII speeds calculated at each time-point. By doing so, we minimized aberrant alterations that might occur specifically either in early or late times of this transcription run-off assay, as previously reported (29).

### Transcription run-on

Run-on assays were performed as previously described (23) with minor modifications. Basically, 25 ml of yeast culture were collected at OD_600 nm_ 0.5 by centrifugation. Cells were washed twice in 5 ml of 0.5% sarkosyl solution for permeabilization, chromatin clearance and transcription ternary complexes fixation. *In vivo* transcription was performed for 5 min at 30°C by re-suspending cells in an appropriate a transcription mix consisting of transcription buffer (50 mM Tris/HCl pH 7.7, 500 mM KCl, 80 mM MgCl_2_), ribonucleotide mix (ATP, GTP and CTP of 10 mM each), 0.1 M DTT and [α-^32^P]UTP (3000 Ci/mmol). The reaction was stopped by adding 1 ml of cold water and RNA was immediately extracted following the acid-phenol protocol. Slot-blotted membranes were elaborated as formerly described (28), with double-strand immobilized probes previously obtained by PCR using primers listed in Supplementary Table S2. The phosphorimaging analyses were performed in a STORM-840 imaging system (GE Healthcare) and quantified using GelQuant.NET software provided by www.biochemlabsolutions.com. All run-on signals were normalized to radiolabelled DNA signals and to the number of cells harvested in each mutant.

### Western blot

Laemmli-boiled crude extracts were run on a SDS-polyacrylamide gel and transferred to nylon membranes (Hybond-ECL). After blocking with Tris-buffered saline containing 0.1% Tween 20 and 5% milk, the following primary antibodies were used: mouse monoclonal anti-HA 16B12 (ab130275; Abcam), mouse monoclonal anti-Rpb3 (ab81859; Abcam) or mouse monoclonal anti-Pgk1 22C5D8 (Invitrogen). Finally, the membrane was incubated with peroxidase-conjugated goat anti-mouse IgG and proteins were detected using a chemiluminescence detection kit (Super-Signal West Pico, Pierce) and a ChemiDoc™ MP system (Bio-Rad).

### Calculations and statistics

All results were calculated and represented using Microsoft Excel. The statistics were carried out using SPSS. A Student’s *t*-test was used to determine significant differences between biological samples. When correlating variables, a Spearman regression coefficient was carried out using a two-tailed significance test.

The R package BayesVarSel (https://CRAN.R-project.org/package=BayesVarSel) was used to perform a Bayesian analysis of the inclusion probabilities among some of the variables. We used the Bvs function of this package.

Principal components analysis was performed to determine possibly correlated variables or mutants. This was done using the ‘prcomp’ function in R.

### *In silico* experiments

NetLogo in its latest version (currently 6.0.2) (https://ccl.northwestern.edu/netlogo/) was used to program and run the model. SPSS in its latest version to conduct statistical analysis of the results both in experimental data and in *in silico* data obtained with the model.

### ChIP-seq

ChIP of both HA-TFIIS and Rpb3 were performed in parallel with the same cell extract, sharing the same input. Two biological replicates of each strain were done and an untagged strain was used as a control. The resulting DNA was sent to CRG Genomics Unit where libraries were prepared and sequenced in an Illumina HiSeq 2000 instrument to an output of 1713.8 (1 × 50) million reads. The quality metrics of the Fastq sequencing datasets were obtained with FastQC and visually inspected. After de-multiplexing, sequencing adapters were removed from raw reads with Fastx Clipper. Low-quality 3′-end of reads were trimmed with Sickle, and then PCR duplicates were removed with Fastx Collapser to ensure each fragment was represented only once. A second round of inspection with FastQC was carried out after the filtering steps to verify that the filters were applied properly. The remaining high quality sequences were mapped to the SacCer3 genome with Bowtie2, using default parameters. Resulting BAM alignment files were visually inspected with the genome browser IGV (www.broadinstitute.org/igv/). Normalized coverage tracks for genome browser visualization were generated with the functions bamCoverage and bamCompare from the deepTools2 suite. Coverage tracks from individual samples are expressed as reads per kilobase per million mapped reads (RPKM), whereas comparison tracks are expressed as the log2 of the number of reads ratio. Normalized average density plots around genomic features were calculated with the ngs.plot package (30). The ChIP-seq mode and the statistical robustness parameter, which filters out 0.5% of genes with the most extreme expression values, were applied to all calculations. The average occupancy profiles of IP and no-tag samples were compared to each other to verify that both profiles were different. No-tag datasets were not used for any further downstream analysis. Based on the high similarity of the average occupancy profiles from the two biological replicates, the two datasets from each sample were merged into one for average metagene representations around genomic regions with SamTools (31). The ngs.plot tool was also used to generate bell-shaped heat map representations centred around gene midpoints.

### Chromatin-enriched fractions

Chromatin-enriched fraction purification was performed according to the yChEFs procedure (32). Briefly, 150 ml of exponentially growing yeast cells (OD_600_ ∼ 0.7–0.8) were harvested, at least in triplicate. The whole final purified chromatin-enriched fraction P3 was resuspended in 20 μl of 1× Tris-Glycine SDS Sample Buffer and incubated for 5 min at 100°C, followed by spinning at 10 000 rpm for 30 s. This chromatin pellet was used for SDS-PAGE, followed by western blotting with different antibodies: anti-C-Myc (9E10 Santa Cruz Biotechnology), anti-Rpb3 (anti-POLR2C; 1Y26, Abcam), anti-Histone H3 (ab1791; Abcam) or anti-phosphoglycerate kinase, Pgk1 (459250; Invitrogen). Histone H3 was used as a control of purified chromatin and Pgk1 as a control of cytoplasmic contamination.

## RESULTS

### *GAL1* [mRNA] resists most perturbations of synthesis and decay

Simultaneous analysis of mRNA synthesis and decay is difficult, since most methods for measurement of mRNA half-life involve stressful inhibition of global cell transcription or incorporation of nucleotide analogues that can affect both transcription and mRNA stability. *GAL* genes can be especifically shut-off by adding glucose. We decided to use *GAL1* as a suitable model to study the effect of mRNA degradation mutants on transcription elongation and vice versa. We studied the response of *GAL1* to the genetic perturbation of mRNA decay and transcription elongation machineries by measuring parameters that define the different levels of the gene expression pathway. We studied mutants lacking Xrn1, the main cytoplasmic 5′-3′ exoribonuclease (33); Ccr4, the main cytoplasmic deadenylase and a subunit of the Ccr4–Not complex (10); Not4, another component of this complex (34) that controls de ubiquitylation and degradation of arrested RNAPII (35); Dhh1, an RNA helicase involved in several steps of the mRNA cycle that is tightly associated with both de decaysome (9,22) and Ccr4–Not complexes (36,37); Dst1/TFIIS, a transcription factor able to stimulate the resolution of RNAPII backtracking events (38); Sfp1, a transcription factor that stimulates expression of most growth-related genes and that functionally interacts with TFIIS (39); and Pfd1, a subunit of the prefoldin complex that contributes to chromatin dynamics during elongation and also displays a strong functional interaction with TFIIS (40). We also included a point mutant of Rpb1 (*rpb1-I756A*), in which we have changed an amino acid involved in the interaction between the 3′ end of pre-mRNA and a conserved domain of RNAPII when this is backtracked (41) (Supplementary Figure S1). This mutation does not map in the active centre of RNAPII and, therefore, its phenotypes should not be due to the catalytic defects exhibited by other RNAPII elongation-defective mutants (29,42).

For every mutant, we calculated total RNAPII occupancy of the *GAL1* gene body by anti-Rpb3 ChIP, density of actively elongating RNAPII molecules by transcriptional run-on, and apparent [mRNA] and mRNA half-life by RT-PCR of samples taken several times after adding glucose to cells exponentially growing in galactose (Supplementary Figure S2A). We also calculated RNAPII speed in each mutant using the *GAL1p-YLR454w* system, in which one of the longest genes of the yeast genome is driven by a *GAL* promoter (43) (Supplementary Figure S3A–C). The complete set of values obtained in this study can be found in the Supplementary Data, and a summary of the results for each mutant normalized to the wild-type is shown in Supplementary Figure S2B.

**Figure 1.**
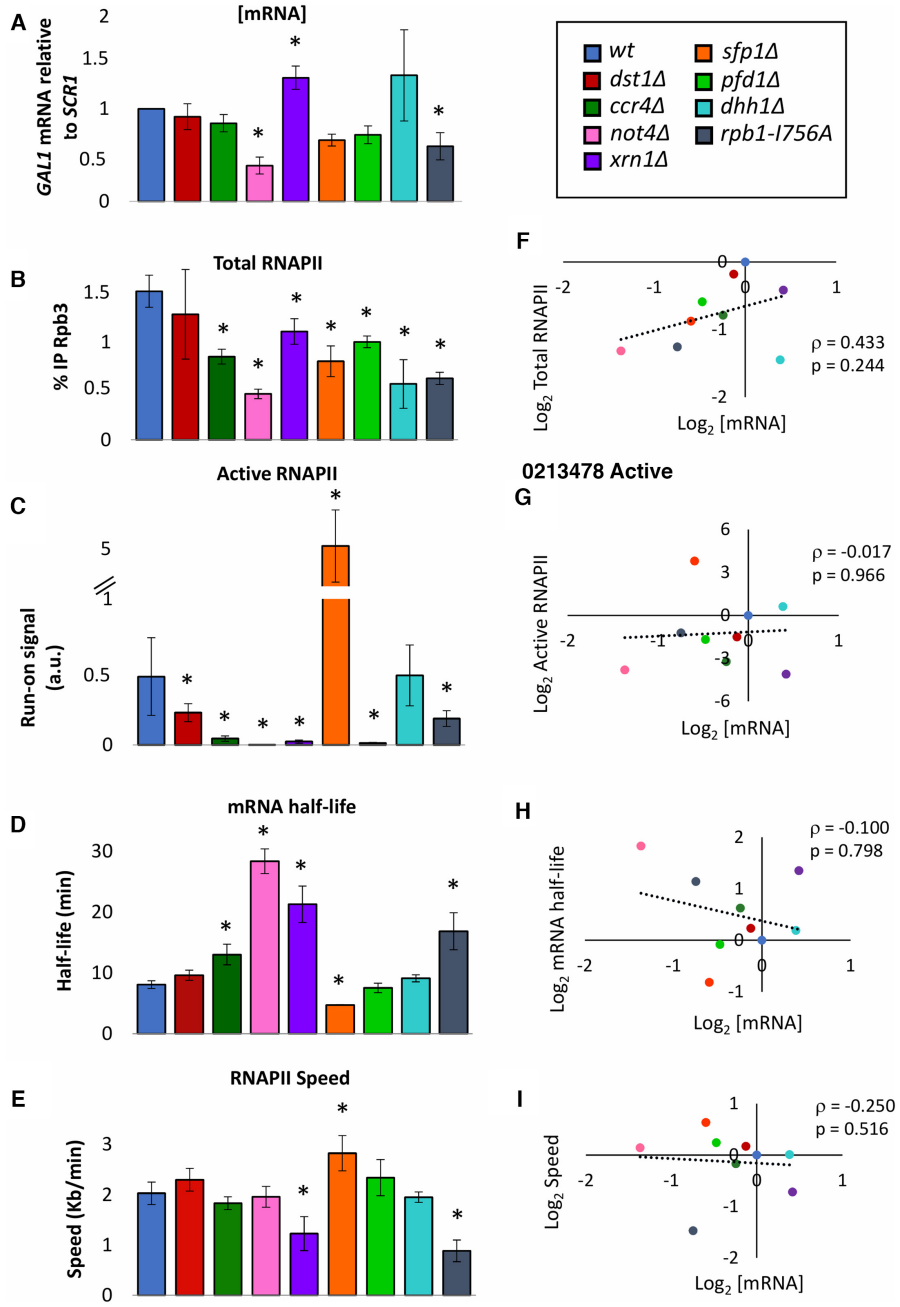
*GAL1* mRNA levels resists most perturbations of synthesis and decay. (**A**) *GAL1* mRNA levels in exponential cultures of the indicated mutant strains. Apparent [mRNA] was calculated relative to *SCR1* by RT-qPCR. (**B**) Total RNAPII occupancy as measured by anti-Rpb3 ChIP. Each bar represents the average value of four amplicons across the *GAL1* gene. (**C**) Active RNAPII as measured by the incorporation of radioactive ^32^UTP (transcriptional run-on). The bars represent the average value of four probes across the *GAL1* gene. (**D**) *GAL1* mRNA as measured by RT-qPCR before and after shutting off transcription by glucose. Half-lives were determined by calculating the time it takes for half of the initial mRNA to be degraded. (**E**) RNAPII speed as measured by anti-Rpb3 ChIP in the long GAL1p-YLR454w gene before shutting off transcription, and three times afterwards. Values were normalized to time 0, and RNAPII speed was calculated. All bars represent mean and standard deviation of three biological replicates (* if *p* < 0.05 in a Student’s *t*-test). (**F**–**I**) To the right of each of the previous graphs, we have represented the corresponding variable with respect to apparent [mRNA]. We never found any significant correlation using a Spearman’s test (*ρ* represents the Spearman coefficient and the *p*, the *p*-value).

Significant changes in RNAPII occupancy, active RNAPII density and mRNA half-life where found in most mutants analysed. However, apparent [mRNA] levels were much less affected indicating a robust homeostasis in most cases (Figure 1A–E). Only *xrn1Δ, not4Δ* and *rpb1-I756A* exhibited significantly altered apparent [mRNA] suggesting that Xrn1, Ccr4–Not and the backtracking-related domain of RNAPII are particularly relevant for mRNA homeostasis (Figure 1A). In fact, *xrn1Δ* and *rpb1-I756A* were the only two mutants affected in all the parameters measured, including RNAPII speed (Figure 1E).

Very similar results were obtained when we analysed the *GAL1p-YLR454w* gene (Supplementary Figure S4A–D). In this case, however, apparent [mRNA] was also significantly decreased in *dst1Δ, ccr4Δ, pfd1Δ* and *sfp1Δ*, which was expected for mutants functionally linked to RNAPII elongation and that strongly affect the expression of long genes (40,44).

We wondered if [mRNA] was preferentially associated with any of the other four parameters measured in this work. We plotted apparent [mRNA] of *GAL1* in each strain against mRNA half-life, total RNAPII occupancy, active RNAPII and RNAPII speed values, and we looked for statistical association. No significant correlation was found after Spearman’s test between [mRNA] and any of the four parameters analysed, demonstrating that none of them predicts [mRNA] on its own (Figure 1F–I).

Since no correlation was detected so far, we wondered whether our data were too noisy due to technical reasons. We decided to analyse the correlation between synthesis rates, formulated as the density of nascent transcription divided by cell volume (see (22) for details) and degradation rates, calculated from apparent [mRNA] and mRNA half-life. We found a very significant correlation between these two parameters in our set of mutants (Supplementary Figure S5A). This correlation was improved even more when RNAPII speed was considered for the calculation of synthesis rates (Supplementary Figure S5B). Interestingly, the correlation was further enhanced when *xrn1Δ* or *rpb1-I756A* were removed from the data (Supplementary Figure S5C). Altogether, these results confirm that alterations of mRNA synthesis can be to a large extent compensated at the degradation step and vice versa.

We have previously described that cell volume influences nascent transcription and mRNA degradation rates (45). To test if our data reproduced this observation, we calculated the correlation between mRNA half-life and cell volume. We found a significant positive correspondence (Supplementary Figure S6A). We also found a negative correlation between the density of active RNAPII molecules (transcription run-on signal) and the cell volume (Supplementary Figure S6B). We have also described that growth rate stimulates global transcription (46). Accordingly, we found a positive correlation between the total amount of RNAPII bound to *GAL1* in each strain to the respective growth rates (GR) (Supplementary Figure S6E). However, total RNAPII occupancy (Rpb3 ChIP signal) did not correlate with cell volume (Supplementary Figure S6C); and active RNAPII did not correlate with GR (Supplementary Figure S6C and D). This suggests a regulatory link between GR and gene transcription at the level of PIC formation, which limits the number of RNAPII molecules transcribing the gene, whereas the connection between transcription and cell volume would be mediated by the fraction of actively elongating RNAPII molecules. On top of these suggestions, the correlations detected confirmed that our data are not noisy.

### RNAPII elongation parameters correlate with mRNA half-life

**Figure 2.**
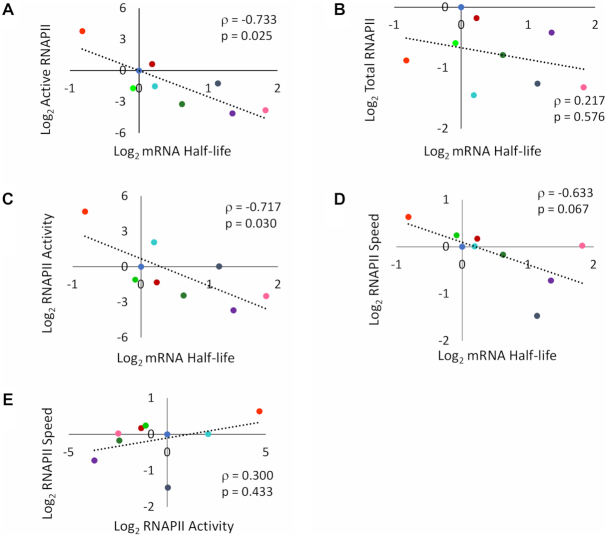
Speed and activity of elongating RNAPII correlates with mRNA half-life. Scatter plots comparing normalized values of the mutants mentioned in Figure 1 for active RNAPII versus mRNA half-life (**A**), total RNAPII occupancy versus mRNA half-life (**B**), RNAPII specific activity (active/total ratio) versus mRNA half-life (**C**), RNAPII speed versus mRNA half-life (**D**) and RNAPII speed versus RNAPII specific activity (**E**). Plots (A), (C) and (D) all showed negative correlations, whilst (B) and (E) presented no significant correlation. In all graphs, the *ρ* represents the Spearman coefficient. *P*-values are also shown. Colour code like in Figure 1.

The correlations of cell volume with mRNA half-life (positive, Supplementary Figure S6A), and active RNAPII levels (negative, Supplementary Figure S6B) predict a negative correlation between the latter. We found it indeed (Figure 2A). In contrast, we found no correlation between mRNA half-life and total RNAPII occupancy (Figure 2B). The ratio between active and total RNAPII (RNAPII specific activity) also negatively correlates with mRNA half-life (Figure 2C). Since the differences between active and total RNAPII molecules have to be caused by inactivation (likely by backtracking) during elongation, these results suggest a link between mRNA synthesis and degradation at this step of transcription.

Co-transcriptional mRNA imprinting has been shown to connect mRNA synthesis and decay (4,47). It has been proposed that RNAPII backtracking might modulate this imprinting (7). Increased backtracking has been shown to decrease RNAPII speed (19). Thus, we looked for and found a negative correlation between RNAPII speed and mRNA half-life when using data from all mutants (Figure 2D). Since RNAPII speed (negatively) and mRNA half-life (positively) both correlated with cell volume (Figure 2D and Supplementary Figure S6F, respectively), we performed Bayesian analysis to rule out the possibility that the correlation between speed and mRNA half-life was mediated by cell volume. Speed and volume together explained mRNA half-life better than cell volume alone, while cell volume is better explained by half-life alone (Supplementary Table S3).

We have shown that growth rate stimulates mRNA decay (46). In fact, reduced mRNA stability in some RNAPII mutants is due to their limited growth rate (29). We wondered if the negative correlation between RNAPII speed and mRNA half-life was due to growth alterations. However, we found no correlation between RNAPII speed and growth rates (Supplementary Figure S6G).

Negative correlation of mRNA half-life with RNAPII specific activity was higher than with speed, due to the outlier behaviour of *not4Δ* and *rpb1-I756A* data points. *not4* lacks a subunit of the Ccr4–Not complex, which has been shown to mediate mRNA imprinting (7). It decreased activity without affecting speed. *rpb1-I756A* decreased speed without affecting activity. As a consequence of these and other mutants, RNAPII speed and specific activity did not correlate (Figure 2E). Our RNAPII elongation speed measurements are based on ChIP experiments with the *GAL1p-YLR454w* system after promoter shut-off. Since this system can be confounding in some specific mutants (29), lack of correlation between RNAPII speed and specific activity should be considered cautiously. Even so, our results suggest that the functional connection between transcriptional elongation and mRNA decay is complex and might involve other phenomena in addition to mRNA imprinting.

### Multi-agent modelling predicts an additional regulatory connection from mRNA decay to transcription elongation mediated by Xrn1

Physical interaction of Xrn1 to promoter regions has been reported and Xrn1 has been proposed to activate PIC assembly and/or RNAPII initiation (9). Accordingly, the transcriptional phenotypes of *xrn1Δ* might be a combination of its direct effect on initiation and an indirect effect on elongation, mediated by Ccr4–Not, the only mRNA decay factor that has been demonstrated so far to stimulate RNAPII elongation (11). We constructed a multi-agent model based on these functional interactions (Supplementary Figure S7A). We defined the properties of each agent (gene, RNAPII, mRNA, TFIIS, Ccr4–Not, Xrn1) and constructed an *in silico* model using the NetLogo programming language (see Supplementary Data: Multi-agent models). Properties of each agent are described in the corresponding flow charts (Supplementary Figure S7B). All agents move and interact in a 3D space divided in two concentric compartments that represent the nucleus and the cytoplasm (Supplementary Figure S7C, Supplementary Data: 3D-model video). Gene, polymerases and TFIIS are confined in the nucleus, whereas mRNAs can go out of the nucleus and be degraded in the cytoplasm. Xrn1 and Ccr4–Not can move to the nucleus by roaming after they finish degrading mRNAs in the cytoplasm (Supplementary data: Multi-agent models). A single gene and six RNAPII molecules were established. The relative number of molecules of TFIIS, Ccr4 and Xrn1 was adjusted according to the information available in the literature, in comparison to the molecules of RNAPII (Supplementary Data: Multi-agent models). These parameters can be modified in the interface, allowing us to test the effect of eliminating any factor (*in silico* mutant) (Supplementary Figure S7D). Some other parameters that are specific of each agent (radius of action, probability of RNAPII backtracking or to reactivate spontaneously) can also be set in the interface. A number of monitors describe in real time the state of the system: fraction of time that the gene is occupied by an RNAPII; mean time (number of ticks) needed by a RNAPII to complete a transcription cycle; proportion of time that elongating polymerases are backtracked; mRNA half-life; average number of mRNA molecules (Supplementary Figure S7D). The evolution of the latter over time can be visualized in a window of the interface (Supplementary Data: computational experiment video). This allows confirming in every experiment one of the expected emerging properties of the system: a steady state number of mRNA molecules, where synthesis and degradation rates are equivalent (Supplementary Figure S7D).

**Figure 3.**
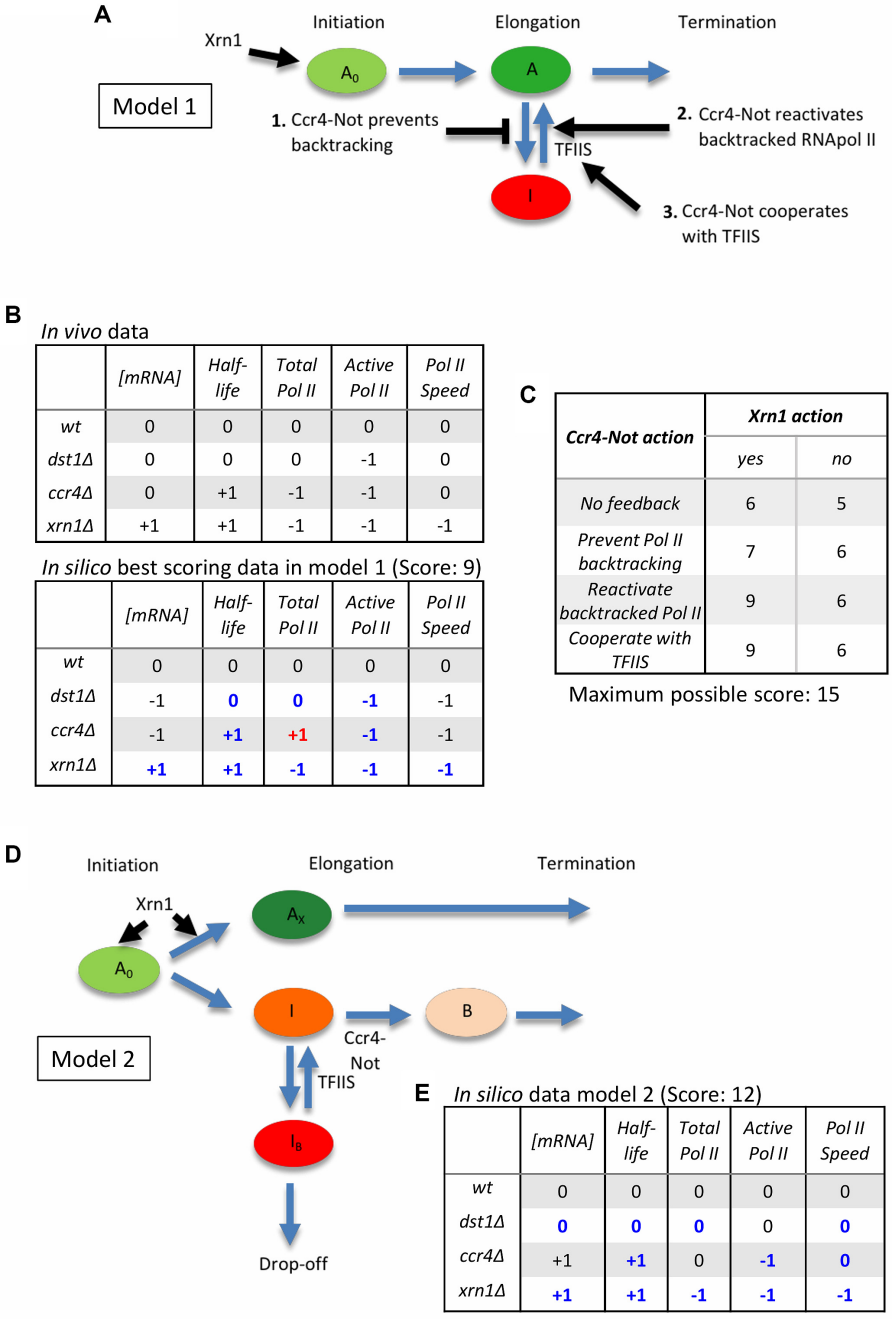
Multi-agent modelling predicts an additional connection from mRNA decay to transcription elongation. (**A**) Scheme showing the possible interactions of the degradation machinery with the transcription machinery conforming the different model variants. An arrow indicates that Xrn1 can stimulate RNAPII initiation. Other arrows indicate Ccr4–Not action where it can either (i) prevent backtracking, (ii) reactivate backtracked RNAPII or (iii) cooperate with TFIIS in reactivating backtracked RNAPII. TFIIS has also been included in this model. It reactivates backtracked RNAPII. (**B**) Matrices showing the *in vivo* data and the *in silico* data for one of the best scoring model variants (Ccr4–Not reactivates backtracked RNAPII and Xrn1 presents feedback to the promoter of genes). We compared total mRNA, mRNA half-life, Total RNAPII, Active RNAPII and RNAPII speed in the *dst1Δ, ccr4Δ* and *xrn1Δ* mutants. If any value was not significantly different from the wild-type, then a 0 was assigned to that variable and mutant. If it was significantly different, then a −1 or +1 was assigned depending on if the number was significantly larger or smaller than that of the corresponding wild-type value. A Student’s *t*-test was carried out to compare values and were considered significant if *p*< 0.05. The hits with respect to the *in vivo* data add 1 point to the overall score. Those values that vary with respect to the *in vivo* data do not change the score, unless it is a −1/+1 difference where 1 point is taken away from the overall score. (**C**) Table showing the scores obtained in the first mathematical model when we compare the model variants to the *in vivo* data. The columns indicate whether Xrn1 presents feedback to the gene promoter (yes) or not (no). The rows indicate the Ccr4–Not action undertaken. The models were scored as indicated in (B), with a maximum possible score of 15. In all cases, there was Ccr4–Not imprinting. (**D**) Scheme illustrating how the second model was programmed. *A*_0_ represents the active RNAPII. Xrn1 can act upon this active RNAPII, turning it into *A*_x_, an RNAPII that does not backtrack and in consequence transcribes faster. If Xrn1 does not act, then the RNAPII can become inactive (*I*). If TFIIS or Ccr4–Not does not act, then the RNAPII turns into a backtracked polymerase (*I*_b_) and eventually drops-off the gene. If TFIIS is present, it can reactivate the polymerase before it drops-off. If Ccr4–Not is present, it can interact with RNAPII and allows it to stay in active transcription. (**E**) Matrix showing the *in silico* data obtained in the second model. Values were assigned as in (B). There were 12 hits with respect to the *in vivo* data, with a total score of 12 over 15.

The first model that we constructed (model 1) involved a positive action of nuclear Xrn1 molecules on transcription initiation, without subsequent action of this factor in elongation (Figure 3A). Regarding the action of nuclear Ccr4, we defined four different variants of the model. In the first case the interaction of Ccr4–Not with an actively elongating RNAPII prevented any backtracking. In the second variant, the interaction of Ccr4–Not with a backtracked RNAPII molecule promoted its reactivation. In the third case, the interaction of Ccr4–Not with a backtracked RNAPII molecule stimulated the recruitment of TFIIS, which in turn reactivated the enzyme (Figure 3A). The fourth and last variant refers to a model where there is no feedback at all by Ccr4-Not. After performing their interaction with RNAPII, Xrn1 can roam and move out of the nucleus to act in mRNA degradation. In the case of Ccr4–Not, it can bind mRNA to be exported together (mRNA imprinting) or can detach from RNAPII at the terminator and move outside of the nucleus (see later). We performed 100 *in silico* experiments and calculated the mean values of five characteristic parameters of the system once it reached steady state mRNA levels, using the information provided by the monitors in the interface: number of mRNA molecules, mRNA half-life, total occupancy of the gene by RNAPII, occupancy by active (non-backtracked) RNAPII molecules and RNAPII speed. We repeated these experiments under conditions of no TFIIS, Ccr4–Not or Xrn1 molecules (*in silico dst1Δ, ccr4Δ* and *xrn1Δ* mutants). We compared the values obtained with the *in silico* wild-type and calculated a data matrix reflecting the differences: 0 meant no significant difference with the wild-type using a Student’s *t*-test (*P* < 0.05), and +1 or -1 meant significantly increased/decreased values, respectively (Figure 3B). A similar matrix was made with the results of the *in vivo* experiments of the *GAL1* gene described above (Figure 3B). We reasoned that the comparison of the two matrices should reflect the similarity between the model and the situation *in vivo*. In order to quantify this similarity, we calculated a score, counting all values that coincided in the two matrices (excluding the wild-type), and subtracting one point in those cases where a -1/+1 difference was found.

Maximal possible score was 15 and the best score obtained was 9, corresponding to the second and third variant of Ccr4–Not action (Figure 3B and C). However, the other variant showed no drastic difference (Figure 3B and C). We also performed similar experiments under conditions where Xrn1 did not have the ability to stimulate initiation. In all variants of the model, lack of Xrn1 action produced some decrease in the global score (Figure 3C), supporting a role of Xrn1 at this level. The minimal score that was obtained was 5, when neither Xrn1 nor Ccr4–Not presented any feedback to transcription elongation (Figure 3C). Therefore, the comparison of *in vivo* and *in silico* results supported transcriptional contributions of mRNA degradation factors. Nevertheless, the highest score obtained was still 6 points short of the maximum possible score of 15. This indicated that this first model was far from fully explaining the transcriptional functions of Xrn1 and Ccr4-Not. Particularly relevant was the observation of RNAPII speed and active RNAPII. In all variants, these two parameters behaved the same, since the only restriction for RNAPII speed in this model was backtracking, which also resulted in lack of activity. However, *in vivo* results showed that *ccr4Δ* and *dst1Δ* caused a decrease in RNAPII activity without significant reduction in speed (Figure 3B). In fact, *xrn1Δ* was the only mutant tested with a significant decrease in speed.

In all variants described above, once Ccr4–Not interacted with the polymerase it remained bound and was transferred to mRNA after termination, simulating an mRNA imprinting process. We repeated the *in silico* experiments, eliminating this mRNA imprinting. The scores obtained were not higher in any case, and in some cases the action of Xrn1 was detrimental (Supplementary Figure S7E). So, lack of fitness of the model was not due to introducing mRNA imprinting.

We decided to design a second multi-agent model with some changes. We distinguished a new state of elongating RNAPII conferred by interaction with nuclear Xrn1 during initiation that prevents backtracking and/or inactivation (Figure 3D). Those polymerases that would not receive this Xrn1 stimulus would be prone to inactivation (giving low activity results in transcriptional run-on experiments) and would be sensitive to backtracking. In addition, the previous RNAPII state would be suppressed by the interaction with nuclear Ccr4–Not, rendering elongating polymerases insensitive to backtracking and less prone to inactivate, although slower than those molecules enhanced by Xrn1 (Figure 3D; Supplementary Data: Multi-agent models). We tested this second model and generated a new matrix of *in silico* results. Comparison with the matrix of *in vivo* results produced a global score of 12, after only three mismatches and no −1/+1 penalty (Figure 3E). Altogether, the results of the multi-agent modelling supported a contribution of Xrn1 to transcription elongation, not mediated by Ccr4–Not.

### *xrn1Δ* and *ccr4Δ* differentially alter RNAPII elongation

**Figure 4.**
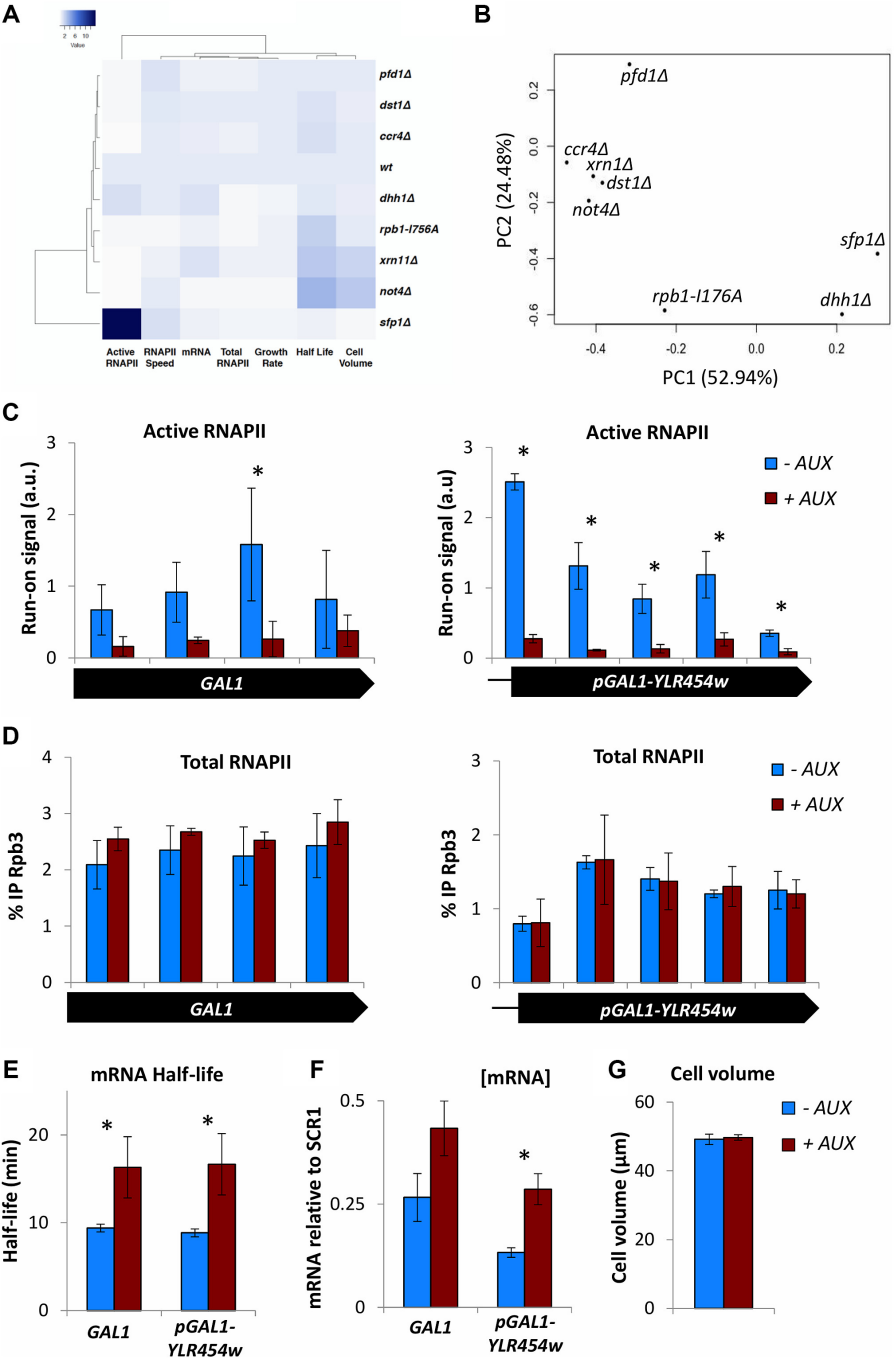
Lack of Xrn1 phenotypically associates with lack of TFIIS and provokes fast inactivation of elongating RNAPII. (**A**) Spearman hierarchical clustering of all the data summarized in Supplementary Figure S2B. *xrn1Δ* primarily associates with *rpb1-I746A*. (**B**) A principal component analysis of the same data associates mRNA decay mutants with *dst1Δ*. The first component (PC1) explains 52.94% of the variables and the second component (PC2) the 24.48%. (**C**) Active RNAPII along the *GAL1* and *GAL1p-YLR454w* gene is decreased in an Xrn1-AID strain, where Xrn1 is rapidly depleted from the cell upon the addition of auxin. Here, we compare the control (-AUX) to Xrn1-depleted cells (+AUX). (**D**) Total RNAPII is unaffected along the *GAL1* and *GAL1p-YLR454w* genes when comparing control to Xrn1 depleted cells. (**E**) The mRNA half-life of *GAL1* and *GAL1p-YLR454w* is significantly increased upon Xrn1 depletion. (**F**) Apparent [mRNA] of both genes is also increased upon Xrn1 depletion. (**G**) Cell volume is unaffected after depleting Xrn1 for 1 h. All bars represent mean and standard deviation of three biological replicates (* if *p*< 0.05 in a Student’s *t*-test).

The multi-agent results prompted us to come back to the experimental evidence showing the involvement of Xrn1 in transcription elongation. First, we performed a clustering analysis of all the experimental data accumulated so far and summarized in Supplementary Figure S2B, based on Spearman correlations. We found that mRNA decay mutants did not cluster together and that *xrn1Δ* primarily associated with *rpb1-I756A*, a mutant specifically affected in the backtracking domain of RNAPII (Figure 4A). We also carried out a principal component analysis of the same data. In this case *ccr4Δ, not4Δ* and *xrn1Δ* grouped, but they did it together with *dst1Δ*. In fact, *xrn1Δ* and *dst1Δ* were the closest mutants in this group (Figure 4B). Thus, two different analyses of the experimental data support a functional connection of Xrn1 to transcription elongation and RNAPII backtracking, suggesting that Xrn1 may regulate transcription by altering elongation rates.

Some mutants causing transcription elongation defects are hypersensitive to NTP-depleting drugs, like 6-azauracil (6AU) (48). We tested sensitivity to different concentration of 6AU for all mutants analysed in this work. As described, we found that *dst1Δ, ccr4Δ* and *not4Δ* were hypersensitive to 50–100 μg/ml 6AU (Supplementary Figure S8A) (49,50). We also found no 6AU sensitivity for *sfp1Δ* and *pfd1Δ*, as reported (Supplementary Figure S8A) (39,40). *dhh1Δ* showed moderate hypersensitivity, as it was unable to grow at 100 μg/ml 6AU, whereas *xrn1Δ* did not show any decrease in viability in the presence of the drug, at any concentration tested (Supplementary Figure S8A). However, when we combined *ccr4Δ* and *xrn1Δ* in the same strain we found a dramatic hypersensitive phenotype, since the double mutant *ccr4Δ xrn1Δ* was unable to grow at 5 μg/ml 6AU (Supplementary Figure S8A). These data support a contribution of Xrn1 to transcription elongation, in the context of previously reported interactions between *ccr4Δ* and RNAPII elongation mutants (50). In fact, we also found a synthetic interaction between *ccr4Δ* and *rpb1-I756A*. In this case, the single *rpb1-I756A* mutant was as sensitive to 6AU as *ccr4Δ*, but the double *ccr4Δ rpb1-I756A* mutant was drastically hypersensitive to this drug (Supplementary Figure S8B).

6AU hypersensitivity is a meaningful phenotype frequently due to transcription elongation defects. However, it is also connected to transcription initiation defects in some RNAPII mutants (29). In order to further characterize the transcriptional involvement of Xrn1, we looked for more direct molecular evidence. So far all Xrn1 results shown were based on the analysis of deletion mutants, which involved a significant limitation in growth. The single *xrn1Δ* showed 3.0 h doubling time in YPD at 30°C, versus 1.8 h of the wild-type. This growth defect was even more severe in the double *ccr4Δ xrn1Δ* mutant, with a doubling time of 8.6 h. These growth defects usually originate pleiotropic phenotypes that are only indirectly related to the genetic change. In order to exclude these possible side effects, we decided to analyse the effect of Xrn1 fast depletion with an engineered version of Xrn1 that is fused to an AID domain and become rapidly degraded in respond to auxin, a non-metabolized molecule. We treated an Xrn1-AID strain for 1 h with auxin and explored its effect on *GAL1* mRNA synthesis and decay. We found a substantial increase in mRNA half-life and [mRNA], which indicated fast and effective degradation of Xrn1 after adding the drug. We also detected decreased density of active RNAPII molecules on *GAL1*, down to <20% of the untreated value, without a significant change in total RNAPII occupancy (Figure 4C–F). Very similar results were obtained for *GAL1p-YLR454w* (Figure 4C–F). No change in cell volume was detected at this time, which indicates that the transcriptional downregulation induced by Xrn1 depletion was not an indirect consequence of cell volume alteration (Figure 4G). These results confirmed a functional link between Xrn1 and RNAPII activity during elongation.

**Figure 5.**
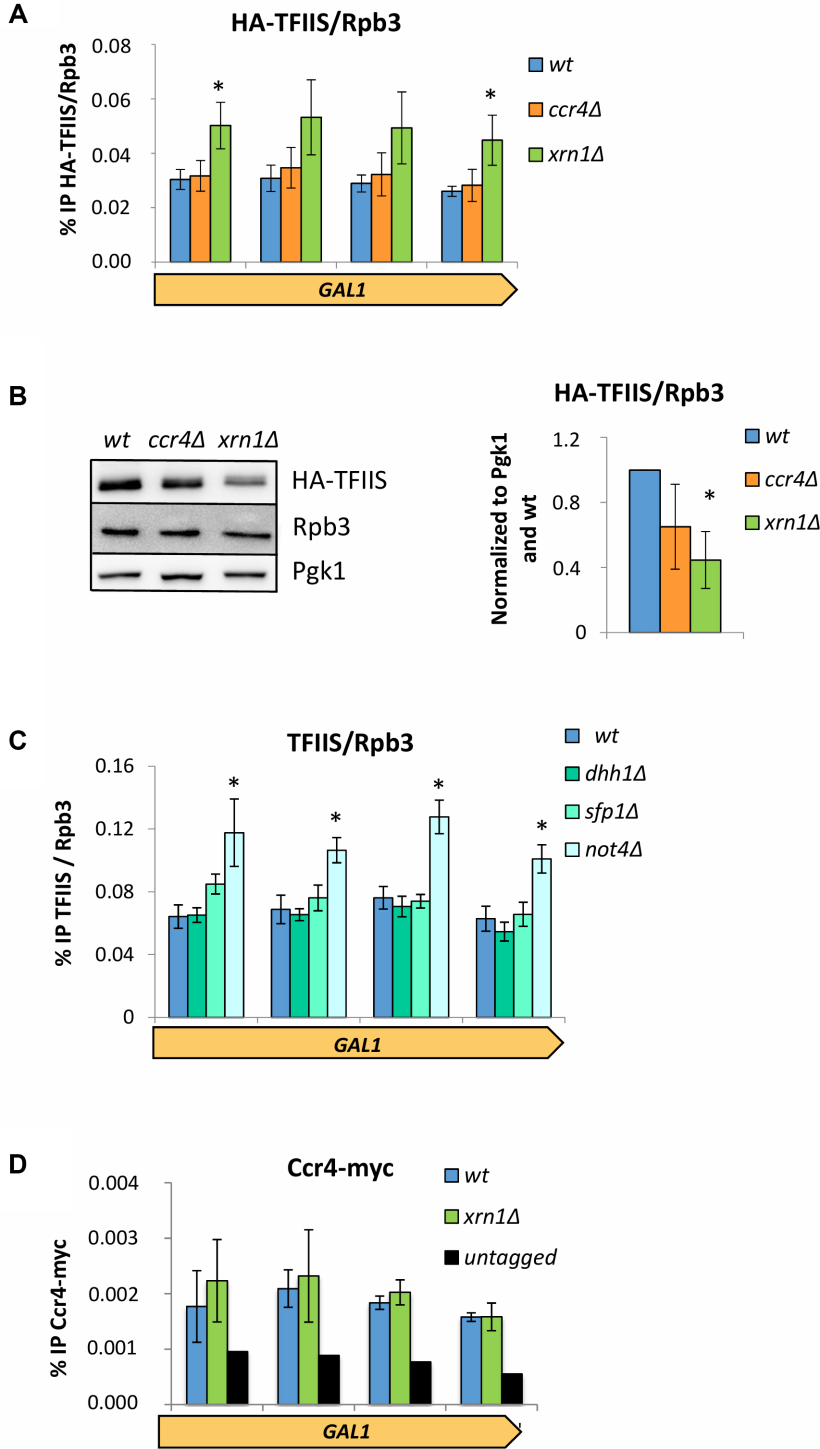
*xrn1Δ* and *ccr4Δ* cause different effects on TFIIS recruitment to elongating RNAPII. (**A**) The amount of TFIIS/Rpb3 found on the *GAL1* gene is significantly increased in an *xrn1Δ* mutant. This was not the case for *ccr4Δ*, as the values were similar to that of the wild-type. ChIPs were performed for HA-TFIIS and Rpb3 using the same cell extract in parallel. We were then able to directly compare the amounts of TFIIS and Rpb3 in each strain. The bars represent the mean and standard deviation of three biological replicates (* if *p*< 0.05 in a Student’s *t*-test). (**B**) The total protein levels of TFIIS/Rpb3 is reduced in *ccr4Δ* and *xrn1Δ* mutants compared to the wild-type. We performed a western blot of total extract proteins and used an antibody against a HA tagged TFIIS, Rpb3 and Pgk1 (loading control). The bars represent the mean and standard deviation of three biological replicates (* if *p*< 0.05 in a Student’s *t*-test). (**C**) The TFIIS/Rpb3 ratio found on the *GAL1* gene is significantly increased in a *not4Δ* mutant. No changes compared to the wt were observed for other mutants tested: *dhh1Δ* and *sfp1Δ*. Results obtained as in (A). (**D**) Ccr4 occupancy of *GAL1* was not altered by lack of Xrn1. ChIPs were performed using anti-C-Myc antibodies. The bars represent the mean and standard deviation of three biological replicates. Values were small but consistent and were in all cases higher than the untagged control.

Low transcription activity with unchanged levels of total RNAPII density strongly suggests increased RNAPII backtracking. This predicts higher recruitment of TFIIS to transcribing RNAPII molecules. In fact, significantly higher TFIIS/RNAPII ratios were found on *GAL1* in *xrn1Δ*, but not in *ccr4Δ* (Figure 5A; Supplementary Figure S9A and B). This increased recruitment of TFIIS is not due to higher levels of TFIIS in *xrn1Δ* cells (Figure 5B). Other mutants causing low RNAPII occupancy of *GAL1* but no decrease in the activity of elongating RNAPII molecules, like *sfp1Δ* or *dhh1Δ* (Figure 2C), did not exhibit significantly higher TFIIS/RNAPII ratios (Figure 5C; Supplementary Figure S9C and D). In contrast, *not4Δ*, which prevents the proteolytic removal of arrested RNAPII complexes (35) and originates a severe decrease in the activity of elongating RNAPII molecules, like *xrn1Δ* (Figure 2C), did show a marked increase of TFIIS/RNAPII ratios in *GAL1* (Figure 5C; Supplementary Figure S9C and D). This indicates that the TFIIS/RNAPII ratio is not unspecifically due to decreased RNAPII occupancy but is a useful parameter to follow defects in RNAPII elongation.

These results support a regulation of RNAPII elongation in response to mRNA decay alterations that is not mediated by Ccr4. In fact, no significant changes in the levels of Ccr4 present in chromatin were detected in *xrn1Δ* (Supplementary Figure S10A). And no reduction in Ccr4 occupancy of *GAL1* was found (Figure 5D). If any, some light increase in the Ccr4/Rpb3 ratios on *GAL1* seems to happen in *xrn1Δ* (Supplementary Figure S10B).

**Figure 6.**
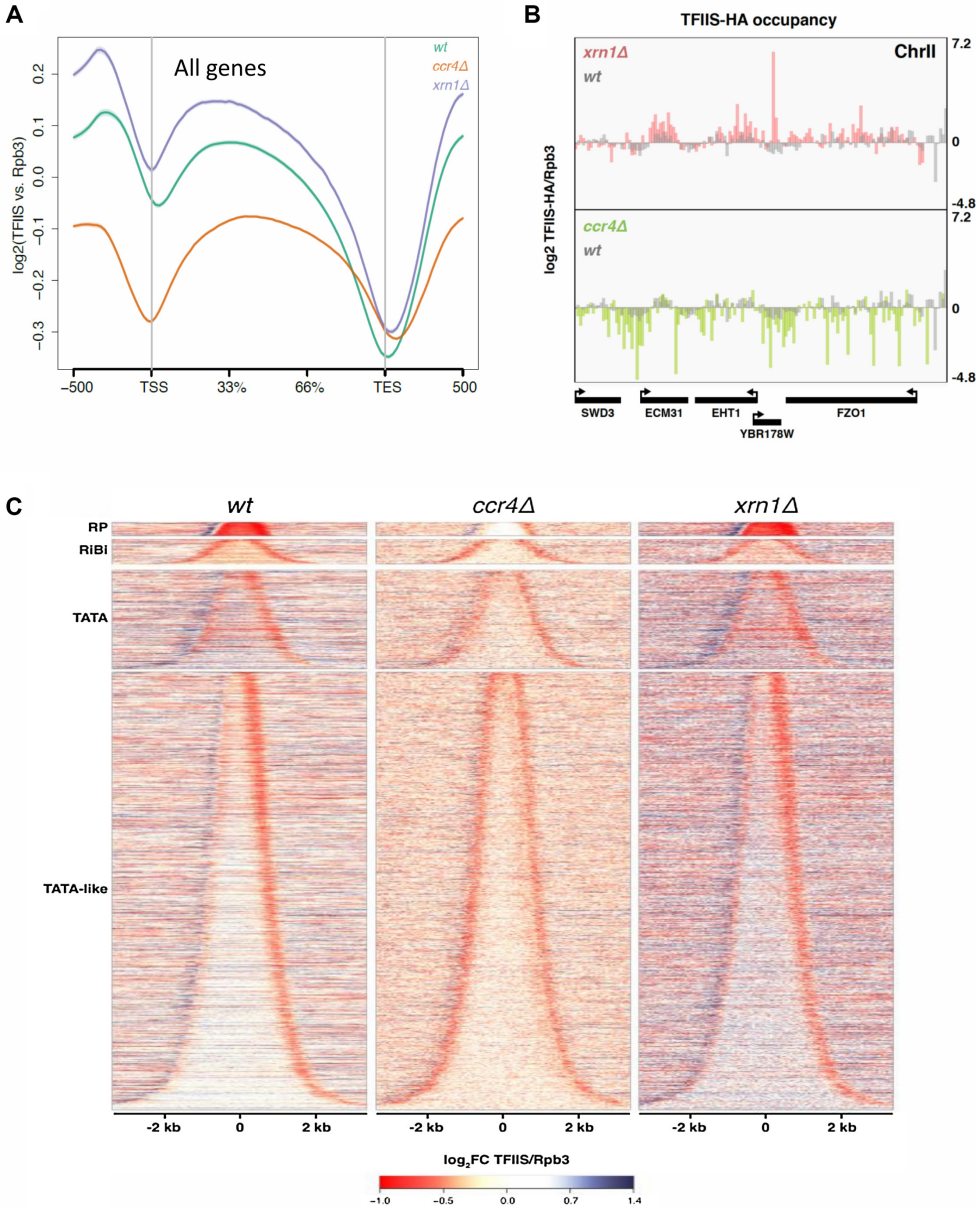
The mRNA decay mutants *ccr4Δ* and *xrn1Δ* show distinct alterations of TFIIS/Rpb3 along genes in a genome wide analysis. (**A**) Whole genome metagene analysis shows that *ccr4Δ* and *xrn1Δ* present different TFIIS/Rpb3 profiles. Lines represent the mean profile of TFIIS/Rpb3 along all yeast genes in wild-type, *ccr4Δ* and *xrn1Δ* strains. TSS is the transcription start site of genes and TES the transcript exit site. The profile is represented as the log2 of the fold change between the TFIIS ChIP and the Rpb3 ChIP. (**B**) The mapping of TFIIS/Rpb3 in five different genes shows an opposite effect for *xrn1Δ* and *ccr4Δ*. There is an increased signal for *xrn1Δ* than for the wild type in this region of the genome. However, when we turn our attention to *ccr4Δ*, we can observe the opposite in the same positions. (**C**) A genome wide study (ChIP-seq) of TFIIS/Rpb3 on genes shows a different profile for *ccr4Δ, xrn1Δ* and the wild type. We have represented all yeast genes ordered by gene length, which leads to a bell shape, and separated out four different groups of genes depending on their promotor type (TATA or TATA-like genes, and within the latter, we seperated out ribosomal protein (RP) genes and ribosome biogenesis (RiBi) genes). In the wt we can observe a downregulation of TFIIS/Rpb3 mostly at the 3' end of genes, and an upregulation in the 5' ends. We obtained a similiar profile for *xrn1Δ*, although with a stronger effect at 5' ends. However, in *ccr4Δ* downregulation of TFIIS/Rpb3 occurs at both 5' and 3' ends of genes. ChIPs of HA-TFIIS and Rpb3 were performed using the same cell extract and in parallel. We represent the mean of two biological replicates.

So far, all experiments have been performed with a model gene. In order to extend our conclusions to the whole genome, we analysed TFIIS/RNAPII ratios by ChIP-seq in *xrn1Δ* and *ccr4Δ*. We confirmed overall higher levels of TFIIS/Rpb3 in *xrn1Δ* than in the wild-type, whereas in *ccr4Δ* we detected the opposite effect (Figure 6A and B). Moreover, we detected significant differences in the profiles of TFIIS/Rpb3 ratios along genes. The wild-type displayed an asymmetric profile, with minimum levels in the 3′ end of genes (Figure 6A). This asymmetric profile was particularly clear in genes containing TATA-like motives in their core promoters, and was enhanced in *xrn1Δ*, which showed stronger increase than the wild-type in the 5′ half of the gene bodies (Figure 6C). In contrast, *ccr4Δ* exhibited a symmetric profile, with similarly low ratios in 5′ and 3′ ends (Figure 6A–C). The 5′ effect of *ccr4Δ* was more evident in TATA-like than in canonical TATA genes like *GAL1* (Figure 6C).

**Figure 7.**
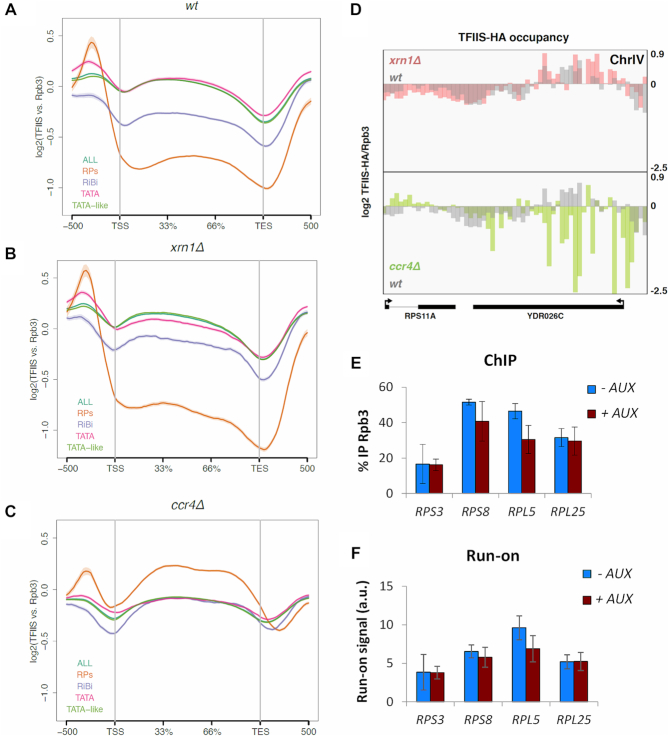
Transcription elongation in RP genes is differentially influenced by Xrn1 and Ccr4, as compared with other gene categories. (**A**) Whole genome metagene analysis of the wild-type shows changes in the TFIIS/Rpb3 profile depending on the gene category. We have represented with a line the mean profile of TFIIS/Rpb3 along all yeast genes. We then divided up the genes into different categories depending on their type of promoter: TATA or TATA-like genes, excluding ribosomal protein genes (RP) or ribosomal biogenesis genes (RiBi). The RP and RiBi genes seem to behave differently from the rest of the genes, with lower overall amounts of TFIIS throughout the entire metagene. TSS is the transcription start site of genes and TES the transcript exit site. The profile is represented as the log2 of the fold change between the TFIIS ChIP and the Rpb3 ChIP. ChIPs of HA-TFIIS and Rpb3 were performed using the same cell extract and in parallel. We represent the mean of two biological replicates. (**B**) Whole genome metagene analysis of the TFIIS/Rpb3 ChIP in *xrn1Δ*. We have represented the same analysis as in (A) but for the *xrn1Δ* mutant. (**C**) Whole genome metagene analysis of the TFIIS/Rpb3 ChIP in *ccr4Δ*. We have represented the same analysis as in (A) but for the *ccr4Δ* mutant. In this case, RP genes show higher TFIIS/Rpb3 ratios than the rest of genes, and RiBi genes behave the same as all genes. (**D**) The mapping of TFIIS/Rpb3 on the RPS11A and YDR026C genes show a distinct effect for *xrn1Δ* and *ccr4Δ*. In the top part of the panel, we can observe the occupancy of TFIIS/Rpb3 in the wild-type and *xrn1Δ*. For the most part, there is similar occupancy in *xrn1Δ* and the wild-type. However, there is clearly decreased signal for *ccr4Δ* (at the bottom of the panel) at the exact same positions. (**E** and **F**) Xrn1 rapid depletion by auxin did not perturb Rpb3 occupancy (E) or activity (F) in RP genes. We performed ChIP against Rpb3 and transcriptional run-on in the AID Xrn1-degron strain without (-AUX) or with (+AUX) auxin addition for 1 h. No significant differences between samples were detected. These Rpb3 ChIP and transcription run-on experiments are directly comparable as the same yeast culture was used for both experiments. We represent the mean of three biological replicates.

Finally, we analysed TFIIS/Rpb3 ratios in other gene families. In the wild-type, genes encoding factors involved in ribosome biogenesis (RiBi) and ribosomal proteins (RP) exhibited lower TFIIS/Rpb3 ratios than the rest of the genome (Figure 7A). In *xrn1Δ* quantitative alterations were detected for TATA-like, canonical-TATA and RiBi genes, in comparison to the wild-type, but no increase was detected in RP genes (Figure 7B). In contrast, TFIIS/Rpb3 ratios of ribosome-related genes increased in *ccr4Δ*, with RP genes situated clearly above the rest of the genome (Figure 7C). So, TFIIS/Rpb3 ratios of RP genes behaved differently than the rest of the genome in both *xrn1Δ* and *ccr4Δ* (Figure 7D). This is a qualitative difference of RP genes that is not due to its high transcription rate, since the top transcribed quartile of genes (excluding RP genes) exhibited completely different profiles (Supplementary Figure S11A–C).

Since we did not detect a general increase in recruitment of TFIIS to elongating RNAPII on RP genes in *xrn1Δ*, we predicted that Xrn1 perturbation should not affect the activity of elongating RNAPII molecules in this gene category. We tested this prediction by quantifying levels of total RNAPII bound to several RP genes, by anti-Rpb3 ChIP, before and after depleting Xrn1-AID with auxin. We also measured the density of active RNAPII molecules on the same genes, by transcription run-on, in the same cultures. In contrast to *GAL1* and *GAL1p-YLR454w* (Figure 4C and D), we found no significant changes in either total or active RNAPII in *RPS3, RPS8, RPL5* and *RPL25* (Figure 7E and F). This indicates that transcription elongation of RP genes is not impaired by Xrn1 perturbation. In combination with our genome-wide analysis, these results confirmed that Xrn1 and Ccr4 contributes to transcription elongation in a differential manner, exhibited by both their effects on elongating RNAPII molecules and the subset of genes affected.

## DISCUSSION

In this work, we have used *GAL1* as a model gene to carefully investigate the effect of a set of mRNA degradation and transcription mutants on gene expression. This analysis allowed us first to confirm the strong robustness of [mRNA] homeostasis when challenged by genetic perturbations that alter mRNA synthesis or degradation. This analysis allowed us to detect new links between mRNA decay and transcription elongation. It has been described that nascent mRNA molecules associate with Rpb4/7 (5,6) and Ccr4-Not (7) during elongation, conditioning its future translation and subsequent degradation. RNAPII backtracking may influence this process. Correlations detected in our data set uncovered a complex relationship between mRNA half-life, RNAPII activity during elongation and RNAPII speed (Figure 2C and E), suggesting that other regulatory links between mRNA decay and transcription elongation might exist. While mRNA imprinting allows controlling mRNA decay from gene transcription (5,12), this additional links might work in the opposite direction, regulating transcription elongation from mRNA decay.

The simplest hypothesis to explain such putative regulation would involve that one or more components of the mRNA degradation machinery might be able to interact with RNAPII and control its elongation behaviour. We hypothesize that, under conditions of long mRNA half-life due to stochastic oscillations, the subsequent mRNA accumulation would make the decay machinery to be totally engaged in degradation. In this situation, no free factors would be available to migrate to the nucleus and foster transcription elongation. In contrast, under the shortage of [mRNA] provoked by short half-life, the pools of free degradation factors would be higher, allowing them to move into the nucleus and stimulate transcription elongation. A similar model has been proposed for the control of nuclear RNA metabolism (51) but can be extended to mRNA metabolism, provided that degradation factors are able to shuttle between the cytoplasm and the nucleus. At least two mRNA decay factors, Ccr4–Not and Xrn1–decaysome, are able to move to the nucleus and interact with the transcription machinery. Ccr4 stimulates transcription elongation by direct interaction with elongating RNAPII (11,15) and cooperates with TFIIS to rescue backtracked polymerases (17). Our computational multi-agent modelling, however, indicated that these two functions of Ccr4 (mRNA imprinting and enhancement of RNAPII elongation) are not sufficient to fully explain our experimental data. Incorporation of Xrn1–decaysome to the model, assuming its role in fostering elongation, provided a better explanation of the data. Additional functions of these factors, like the role of Ccr4–Not in RNAPII assembly (14), might improve future versions of the model. To the best of our knowledge, the utilization of multi-agent modelling to simulate molecular biology interactions is an original methodological contribution of this work.

We have also previously reported evidence showing a functional link between Xrn1–decaysome and transcriptional elongation. Mutants lacking Xrn1 or containing a catalytically dead allele preferentially affect transcription of long genes and show altered profiles of RNAPII along gene bodies (9,13). However, these phenotypes might be indirectly caused by side effects of the lack of Xrn1 function in mRNA degradation; for instance, by decreasing the pool of nuclear Ccr4–-Not able to act on elongation. Our results indicate that this is not the case. Fast depletion of Xrn1 caused dramatic decrease of elongating RNAPII activity in *GAL1* without diminishing growth or increasing cell volume (Figure 4B–F). The levels of Ccr4 occupancy on gene bodies or Ccr4 in the chromatin fraction did not change in cells lacking Xrn1 (Figure 5C and Supplementary Figure S10A). Moreover, lack of Ccr4 did not produce higher recruitment of TFIIS to elongating RNAPII molecules in *GAL1* (Figure 5A). Genome-wide analysis also showed that lack of Ccr4 did not increase TFIIS recruitment to elongating RNAPII (Figure 5D and 6C). In contrast, lack of Xrn1 did produce higher TFIIS/Rpb3 ratios in *GAL1* (Figure 5A) and across the genome (Figures 5D and 6B), in spite of its lower cellular levels (Figure 5B).

We have previously shown that transcriptional action of Xrn1–decaysome requires its transport into the nucleus (9), and that Xrn1 binds the *GAL1* gene body upon transcriptional induction (13). Xrn1 is also recruited to the gene bodies of osmotic-responsive genes during induction (Paula Alepuz, personal communication). This evidence suggests that Xrn1 action on elongating RNAPII would involve its physical interaction with elongating RNAPII. Proteomic analysis of RNAPII elongation complex has reported such an interaction (52), which is also supported by genetic evidence (53). Both physical and genetic interactions between Xrn1 and other factors directly involved in transcription elongation have been reported. In amongst these factors are the CTD kinases Ctk1 (54,55) and BUR (55,56), the chromatin remodeller Isw1 (57) and the histone modifying complex SAGA (58,59). It is, therefore, conceivable that Xrn1 may foster transcription elongation by favouring the structural transition that the RNAPII elongation complex undergoes between promoter escape and transcription termination (60,61). This transition involves the re-configuration of the of RNAPII/Spt6/DSIF/PAF quaternary complex. An action of Xrn1 at this level would be fully compatible with a parallel role by Ccr4, since this factor binds the Rpb4/Rpb7 module of RNAPII and acts on the emerging transcript (15) and the N-terminus domain of recruited TFIIS (17).

Since Xrn1 is a 5′ to 3′ RNA exonuclease, it is also tempting to speculate that Xrn1 may favour eviction of irreversibly arrested RNAPII molecules by some kind of torpedo mechanism of termination. If this would be the case, Xrn1 would need the previous action of the decapping machinery, which also seems to participate in mRNA decay/transcription cross-talk (9). However, we cannot exclude Xrn1 influences transcription elongation at a distance. From its reported location in promoter regions (9), Xrn1 might indirectly enhance elongation by antagonizing antisense ncRNAs, many of which are shown to accumulate in Xrn1-deficient mutants (62). Efforts should be made to address these hypotheses in the near future.

In our hands, lack of Ccr4 provoked decreased RNAPII activity in *GAL1*, without decreasing RNAPII speed and without increasing TFIIS recruitment. Lack of Xrn1 also caused decreased RNAPII activity in *GAL1*, but with increased TFIIS recruitment and enlengthened elongation rate. These differences indicate that Ccr4 and Xrn1 foster elongation in different manners. Moreover, lack of TFIIS does not significantly decrease RNAPII speed, in spite of its significant impact on nascent transcription rates and processivity (Supplementary Figure S3B) (43,63), and the slowest mutant in our hands, *rpb1-I756A*, did not show decreased RNAPII activity. This suggests that in the context of our experimental conditions backtracking tendency, as it is detected by the run-on assay, is not the main limitation of speed. It may contribute in some cases, but other events like RNAPII/nucleosome interaction, nascent RNA dynamics or even the length of the backtracking movement, might be more rate-limiting for speed. According to this, reduced RNAPII speed and increased backtracking frequency would be two different aspects of the elongation defects of *xrn1Δ*. The success of our second multi-agent model, which is based on this hypothesis, supports this view.

Enhanced RNAPII backtracking of *xrn1Δ* would produce the higher recruitment of TFIIS that we detected in *GAL1* and across the genome. In contrast, lack of Ccr4 did not produce such an effect in either *GAL1* or our global analysis, but a general decrease of the TFIIS/Rpb3 ratios in most genes. This fits with the reported role of Ccr4 in facilitating the recruitment of TFIIS to elongating RNAPII (17).

While lack of Xrn1 originated a general increase in the recruitment of TFIIS along gene bodies in our genome-wide study, lack of Ccr4 caused opposite 5′ versus 3′ changes in the distribution of TFIIS, with special incidence in TATA-like genes (Figure 5D; Supplementary Figure S9A and B). This is consistent with the biased 5′/3′ distribution of active RNAPII reported for *ccr4Δ* in a wide set of highly transcribed genes (28). The differential effect of Xrn1 and Ccr4 is also consistent with their distinct association with the RNAPII elongation complex in a model gene, whereas Ccr4 was reported to specifically bind the 3′ half of the gene body, Xrn1 was found to evenly associate to elongating RNAPII from 5′ to 3′ (52).

Unexpectedly, RP genes behaved differently to most other genes in *ccr4Δ* when classified according to TFIIS/Rpb3 ratio, which increased in this group of genes (Figure 6C). It points to a particularly important contribution of Ccr4 to elongation in RP genes. It has been described that RP mRNAs are also especially prone to be imprinted by Ccr4–Not (7), suggesting that co-transcriptional imprinting and stimulation of RNAPII elongation by Ccr4–Not can be coupled phenomena.

RP genes also behaved differentially with respect to Xrn1, since lack of this factor did not increase TFIIS/ratios in this subset of genes, and fast Xrn1 depletion did not reduce the occupancy or activity of the RNAPII molecules engaged in all RP genes that we tested (Figure 7). Since TFIIS/RPB3 ratios of RP genes are particularly sensitive to Ccr4 alteration, these results contradict once more that Xrn1 acts on elongation in a Ccr4-dependent manner.

This differential response of RP genes to changes in mRNA decay fits with their behaviour in the context of growth rate. We have described that transcription and mRNA degradation rates increase in parallel with growth rates, keeping [mRNA] unchanged, and have proposed that this parallelism is due to the molecular cross-talk between the transcription and mRNA decay machineries (64,65). We have also proposed that RP genes can upregulate their expression with growth rate because they can disconnect from this cross-talk. The specificity of RP genes that we have detected backs this hypothesis.

All the evidence described in this work supports elongating RNAPII as an important target of the cross-talk between mRNA decay and gene transcription, and indicates that, in addition to Ccr4, Xrn1 is an important player of this molecular dialogue.

## DATA AVAILABILITY

Our multi-agent software tools can be download from https://github.com/danther/Transcription-Degradation.

The ChIP-seq data are stored in the the GEO repository (accession number GSE127179).

Data can also visualized in the UCSC browser: http://genome-euro.ucsc.edu/s/jorplan/xrm1_ChIP%2Dseq

## Supplementary Material

gkz660_Supplemental_Files

## Notes

Present address: Victoria Begley, UCL Cancer Institute, University College London, 72 Huntley Street, London WC1E 6BT, UK.

Present address: José E. Pérez-Ortín, SciLifeLab, Department of Microbiology, Tumor, and Cell Biology, Karolinska Institute, Tomtebodavägen 23, SE-171 21 Stockholm, Sweden.
