# Supplementary material for: The mRNA degradation factor Xrn1 regulates transcription elongation in parallel to Ccr4
Source: Nucleic Acids Res. 2019 Aug 8;47(18):9524–41. doi: 10.1093/nar/gkz660 (PMC6765136; doi:10.1093/nar/gkz660)
Supplement: gkz660_Supplemental_Files [file gkz660_supplemental_files.zip › Supplementary figures and models.pdf]

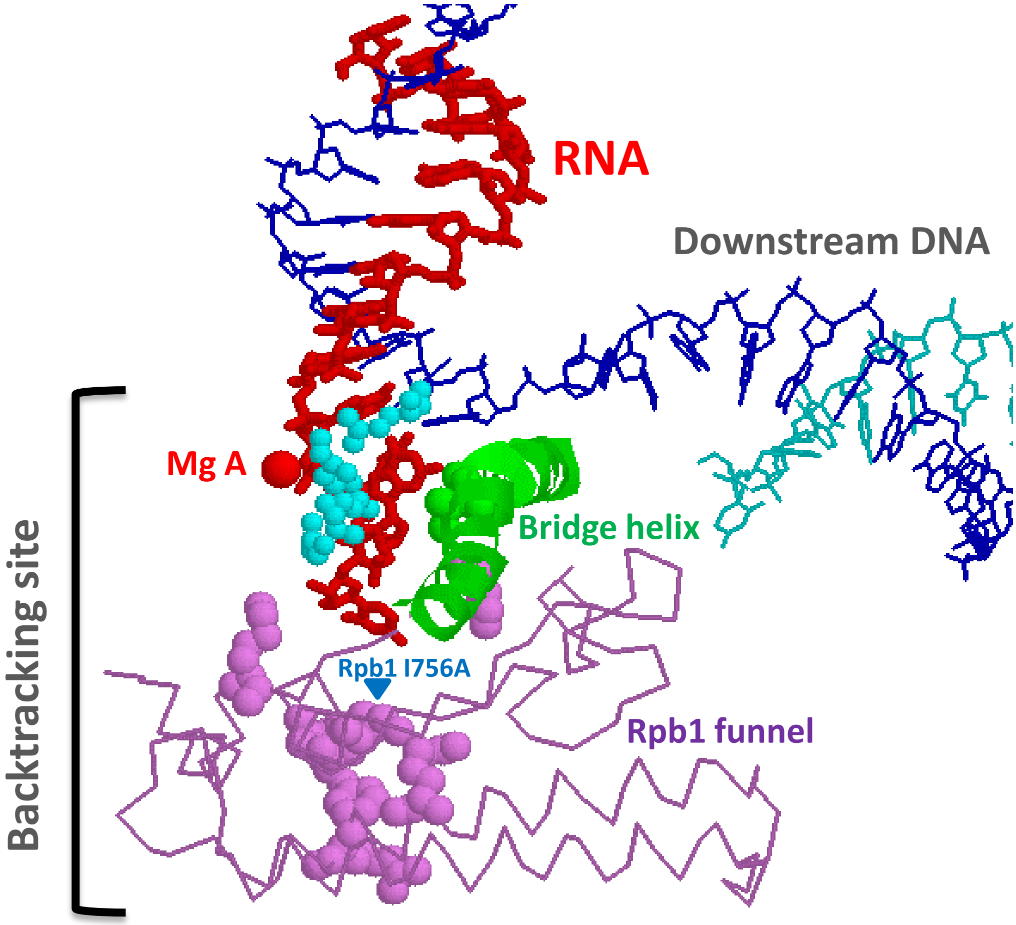

Figure S1: Schematic view of the RNAPII backtracking site and Rpb1-I756A amino acid substitution. Side view of the backtracking site of RNAPII containing RNA (red), downstream DNA (blue and cyan), and different domains of the RNAPII. Spheres show the amino acids considered to contact RNA in the backtracked configuration of Rpb1 (violet and green) and Rpb2 (cyan). Mg A is represented as a single red sphere. Data obtained from (36).

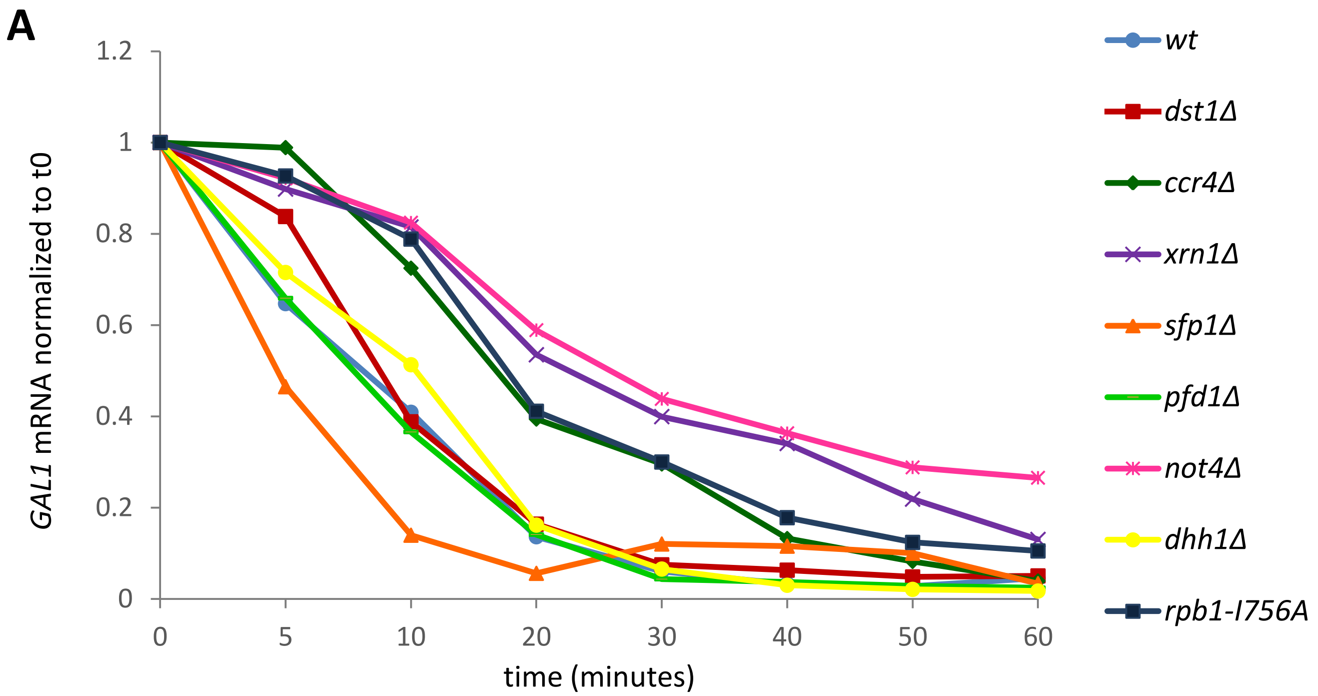

B

	[mRNA]	Half-Life	Total Pol II	Active Pol II	Pol II Speed	Growth Rate	Cell Volume
wt	1.0	1.0	1.0	1.0	1.0	1.0	1.0
dst1Δ	0.9	1.2	0.9	0.4	1.1	0.9	0.8
ccr4Δ	0.8	1.5	0.6	0.1	0.9	0.9	0.9
xrn1Δ	1.3	2.5	0.7	0.1	0.6	0.6	1.9
sfp1Δ	0.7	0.6	0.5	13.9	1.5	0.5	0.4
pfd1Δ	0.7	0.9	0.7	0.3	1.2	1.0	0.9
not4Δ	0.4	3.5	0.4	0.1	1.0	0.4	2.5
dhh1Δ	1.3	1.1	0.4	1.5	1.0	0.5	0.8
rpb1-I756A	0.6	2.2	0.4	0.4	0.4	0.6	1.0

> 10
2.1 – 10
1.3 – 2.0
0.8 – 1.2
0.5 – 0.7
0.2 – 0.4
≤ 0.1

Figure S2. *Q*uantitative effect of mRNA synthesis and decay mutants on *GAL1.* A. Measurements of mRNA stability. *GAL1* mRNA was measured by RT-qPCR before and after shutting off transcription by glucose. Values were normalized to *SCR1*. Eight different time points were taken. B. Summary of all the studied variables in the *GAL1* gene. All the experimental data was simplified into a single representative value with respect to their corresponding wild type values. Aside from [mRNA], half-life, total RNAPII active RNAPII and RNAPII speed, we also studied their growth rates and cell volumes. All values are colour coded to correspond to the map on the right, showing high values relative to the wild type in dark blue, and low values in light blue. The RNAPII speed values were calculated from the experimental data shown in figure S2.

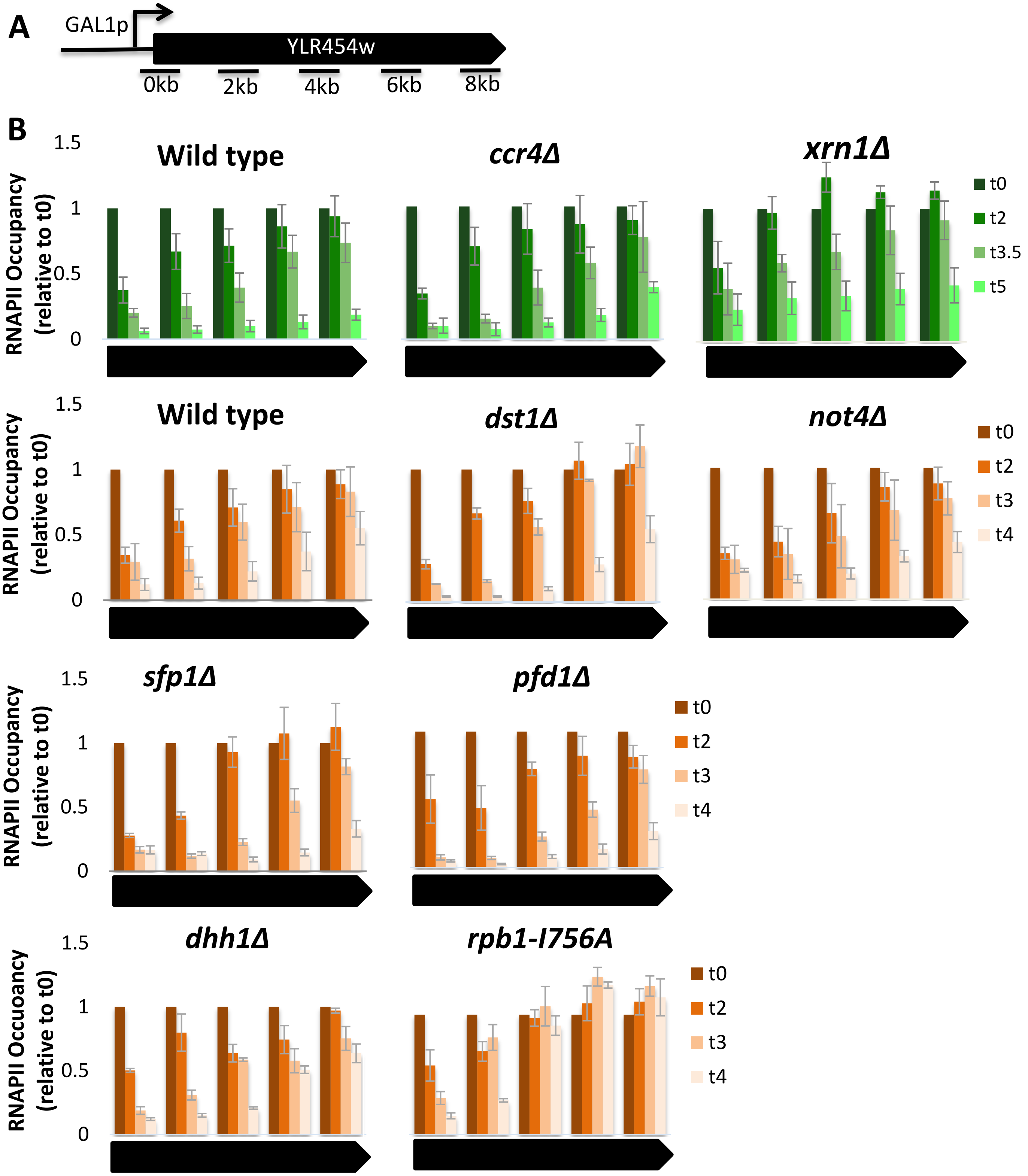

C

wt	**Average Speed (Kb/min)**	**Sd**
1st strech (0-2 min)	1.158	0.405
2nd strech (2-3 min)	2.228	0.732
3rd strech (3-4 min)	3.470	0.840
**Weighted average**	2.004	0.149

Figure S3. Measurements of RNAPII elongation rate in mRNA synthesis and decay mutants. A. Diagram of the long *GAL1p-YLR454w* gene with the annotated positions of PCR amplicons used in B. B. RNAPII speed is significantly altered in *xrn1*Δ*, sfp1*Δ and *rpb1-I756A*. RNAPII levels were measured by anti-Rpb3 ChIP in the long *GAL1p-YLR454w* gene before shutting off transcription (t0), and three times afterwards. Values were normalized to time 0, which is the steady state amount of RNAPII. All mutants were studied in relation to a wild type performed at the same time. Here we show a representative wild type. Each bar represents the mean and standard deviation of three biological experiments. C. The speed at different time intervals were calculated by comparing the area of RNAPII that had abandoned the gene at a given time compared to the previous time. Here we show an example. The weighted average speed was calculated for each strain.

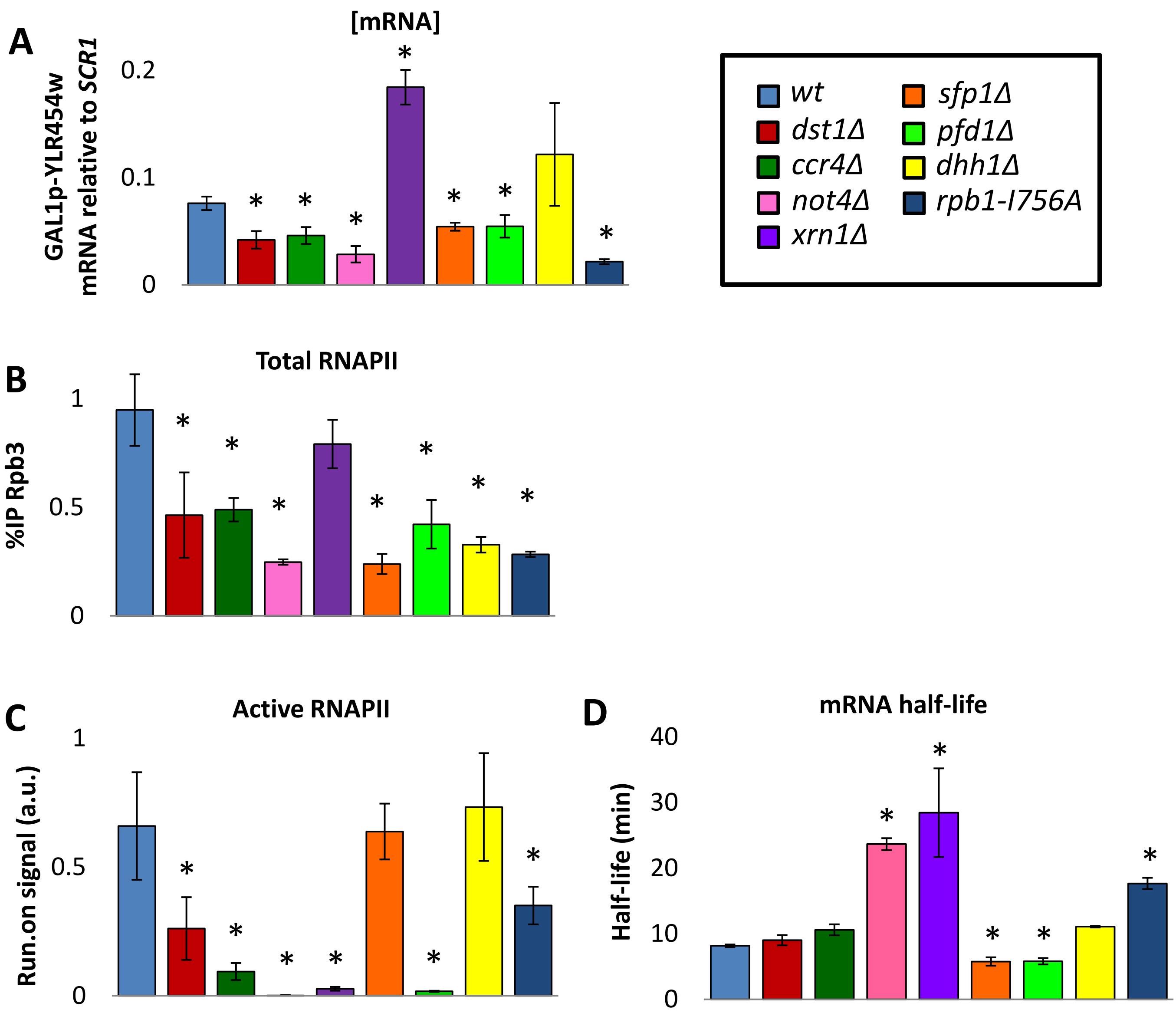

Figure S4. Expression of *GAL1p-YLR454w* is sensitive to most transcription elongation and mRNA decay mutants tested in this work. A. *GAL1* mRNA levels in exponential cultures of the indicated mutant strains*.* [mRNA] was calculated relative to *SCR1* by RT-qPCR. B. Total RNAPII occupancy as measured by anti-Rpb3 ChIP. Each bar represents the average value of four amplicons across the *GAL1* gene. C. Active RNAPII as measured by the incorporation of radioactive ^{32}UTP (transcriptional run-on). The bars represent the average value of four probes across the *GAL1* gene. D. *GAL1* mRNA as measured by RT-qPCR before and after shutting off transcription by glucose. Half-lives were determined by calculating the time it takes for half of the initial mRNA to be degraded. All bars represent mean and standard deviation of three biological replicates (* if p<0.05 in a Student's t-test).

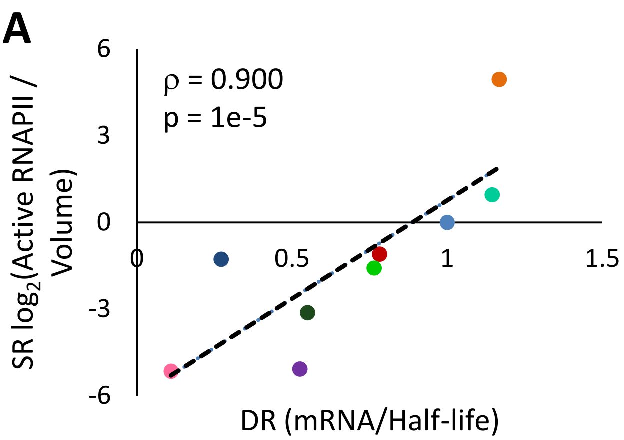

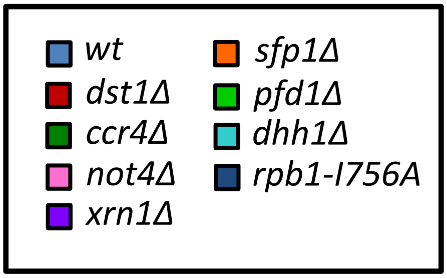

$$\frac{[mRNA]}{HL} \sim \frac{Active\ Pol\ II}{Volume}$$

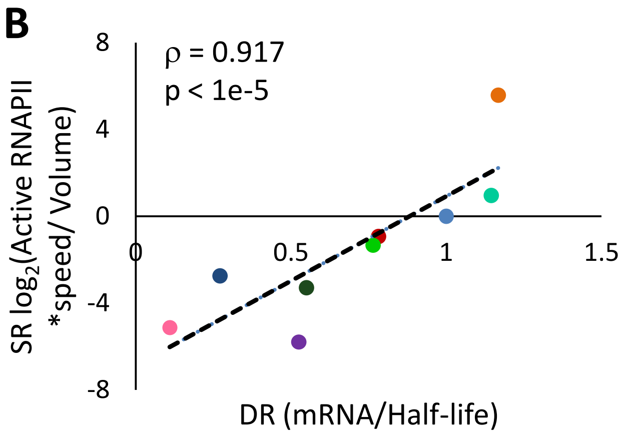

$$\frac{[mRNA]}{HL} \sim \frac{Active\ Pol\ II \times Speed}{Volume}$$

C

Removed	Spearman (ρ)	p-value
none	0.917	0.000
dst1Δ	0.867	0.002
ccr4Δ	0.900	0.001
not4Δ	0.917	0.001
not5Δ	0.917	0.001
xrn1Δ	0.983	0.000
sfp1Δ	0.867	0.002
pfd1Δ	0.867	0.002
dhh1Δ	0.867	0.002
rpb1-I756A	0.950	0.000

Figure S5. Synthesis and degradation rates correlate. A. Plot of synthesis rates (SR) versus degradation rates (DR) calculated as in 22 (mathematical equations shown in the box on the right). Spearman correlation coefficient (ρ=0.900) and significance ($p<0.05$) are shown. B. A new calculation of SR including RNAPII speed led to a better correlation (ρ=0.917). RNAPII speed has been included in the previous SR equation (right) as it was found to contribute to cross-talk in figure 2D. C. There are two potential outliers in the previous equation, *xrn1*Δ and *rpb1-I756A*. We performed a Spearman correlation test after deleting one mutant at a time from the dataset. When *xrn1*Δ was eliminated, the correlation became near to perfect (ρ=0.983). The same occurred when eliminating *rpb1-I756A* (ρ=0.950). All points have been colour coded according to the mutant value they represent.

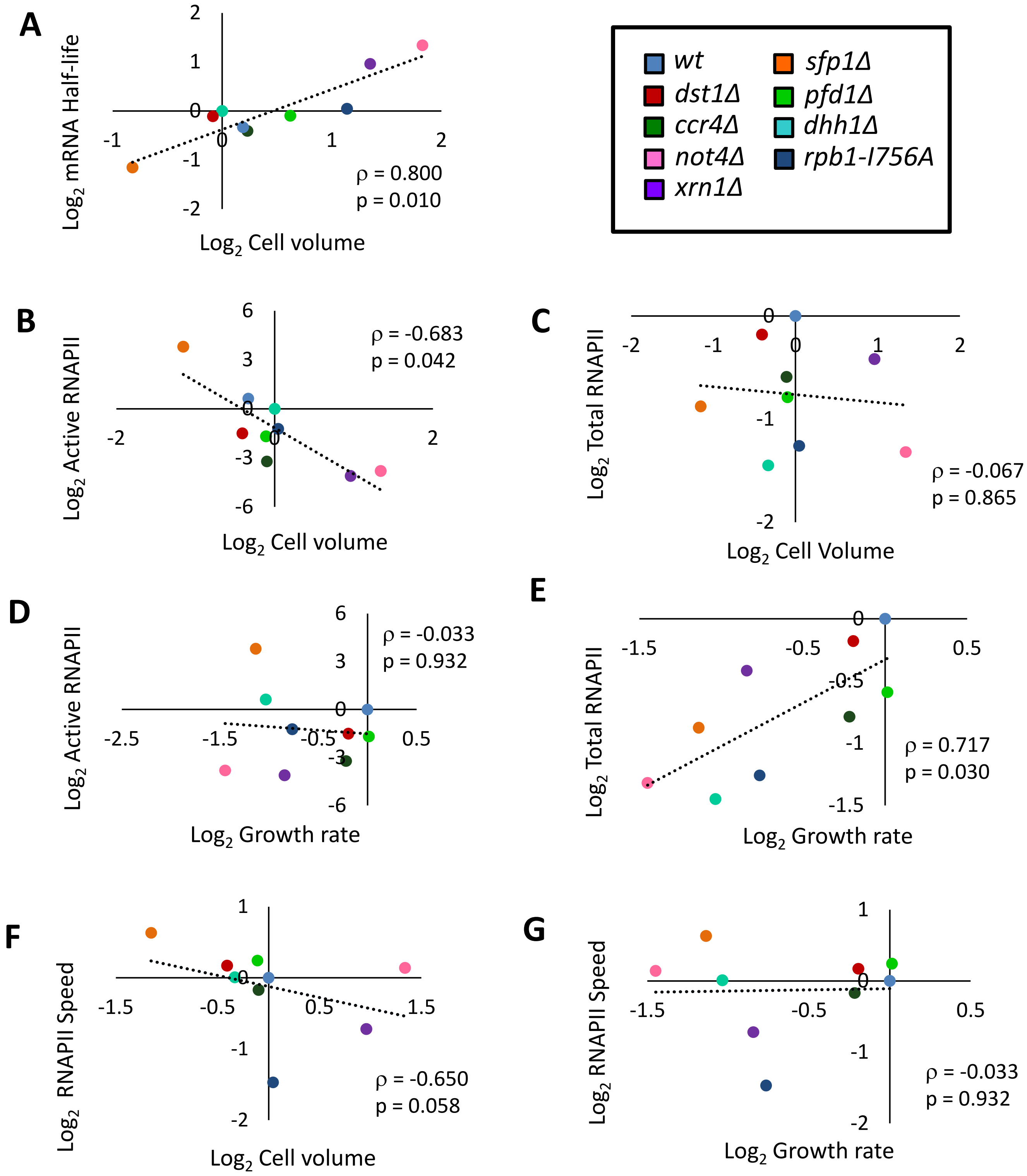

Figure S6. Correlations of transcription and mRNA decay parameters with cell volume and growth rate. A. Cell volume positively correlates with mRNA half-life. B. Active RNAPII, measured by transcriptional run-on, and cell volume negatively correlate. C. Total RNAPII, measured by anti-Rpb3 ChIP, does not correlate with cell volume. D. Active RNAPII does not correlate with growth rate. E. Total RNAPII positively correlates with growth rate. F. RNAPII speed negatively correlates with cell volume. G. RNAPII speed does not correlate with growth rate. In all graphs, the ρ represents the Spearman coefficient and the p the p-value calculated in each correlation. All points have been colour coded according to the mutant value they represent.

A

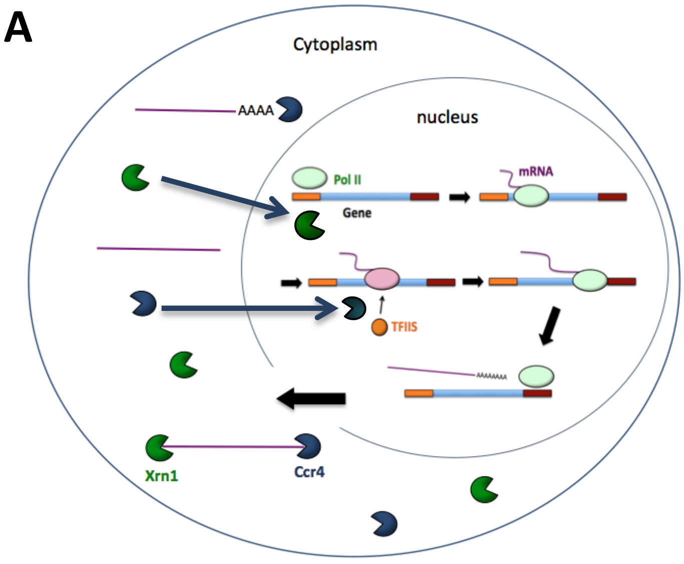

B

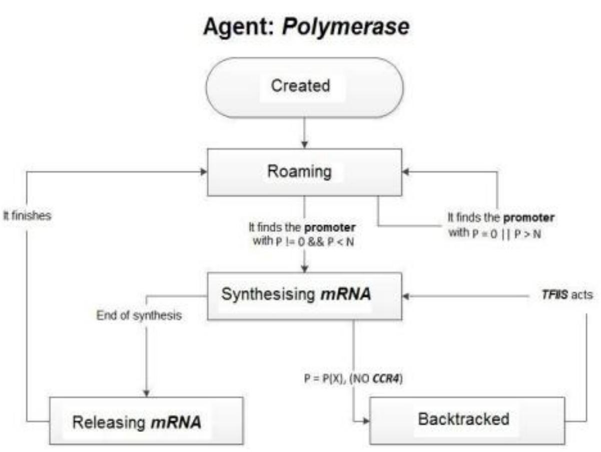

C

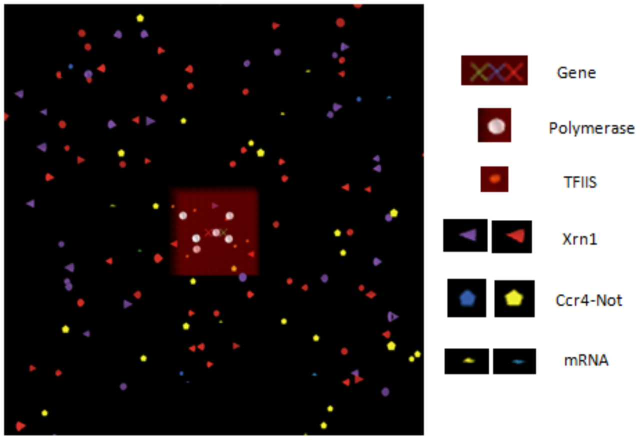

E Scores of model 1 options, without mRNA imprinting.

Ccr4-Not action	*Xrn1 action*	
	yes	*no*
No feedback	6	5
Prevent Pol II backtracking	5	8
Reactivate backtracked Pol II	5	8
Cooperate with TFIIS	6	6

D

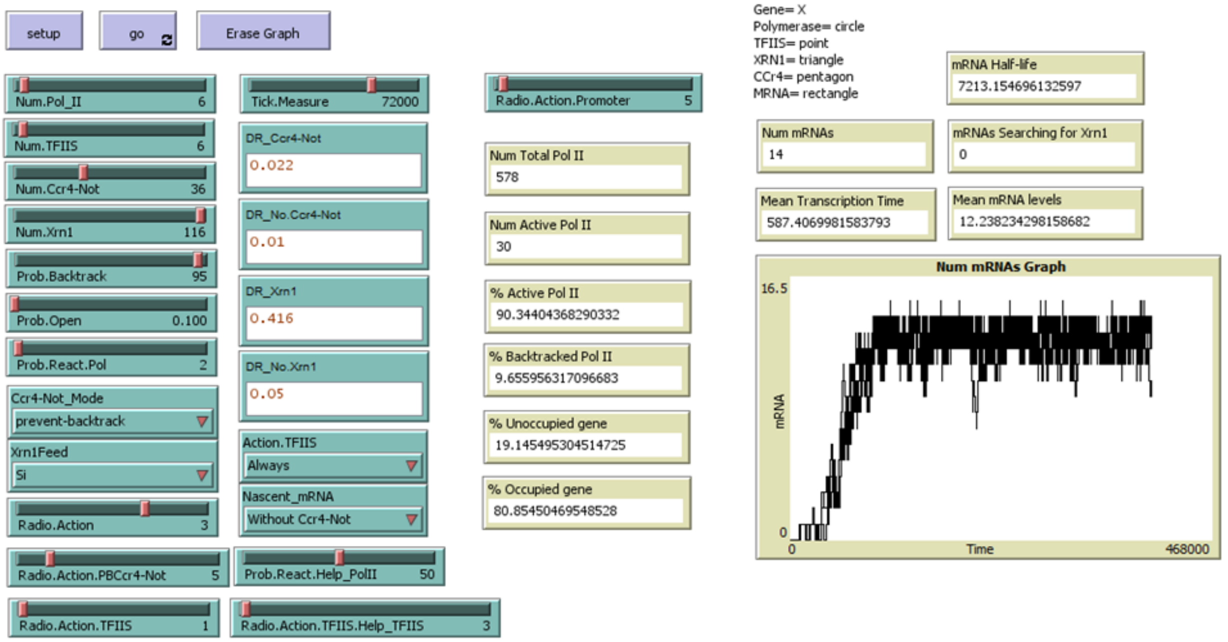

Figure S7. Multi-agent modelling of the crosstalk between mRNA synthesis and decay. A. Scheme of the simplified cross-talk mechanism on which we have based our model. As shown with blue arrows, Ccr4-Not (in blue) and Xrn1 (in green) are able to enter into the nucleus and interact with the transcriptional machinery. In our model they are programmed to do so only after degrading mRNA (purple line) in the cytoplasm. Xrn1 is modelled to interact with the promoter of the gene, and Ccr4-Not with the polymerase either in active (green oval) or in backtracked state (pink oval), or with TFIIS. B. Flow chart for the polymerase. In this flow chart the behaviour of the polymerase is defined as well as its interaction with other agents. Similar flow charts were designed for every agent. C. Snapshots of the simulation world within NetLogo. Snapshot of the 3D version that we are now working with. The nucleus is separated from the cytoplasm and is represented by a red cube. The gene is in the centre of the nucleus, and the rest of the agents are in their corresponding compartments in 3D. The cell is delimited by a bigger black cube. In both cases we can see that the agents are represented by different geometric figures that change colour according to its state. D. NetLogo interface and monitor showing the appearance of one emergent property (steady state of mRNA molecules) after a certain time length. The right graph shows the mRNA levels of 100 different executions of the program. Since the levels always fell into the same range, we can observe the robustness of the system. The green boxes are slider controls enabling us to modify the parameters with ease. In the cream boxes the monitored variables are shown. E. Table showing the scores obtained in the first mathematical model when we compare the model variants to the *in vivo* data. Similar table to 3B, except that in all cases there was no mRNA imprinting by Ccr4-Not.

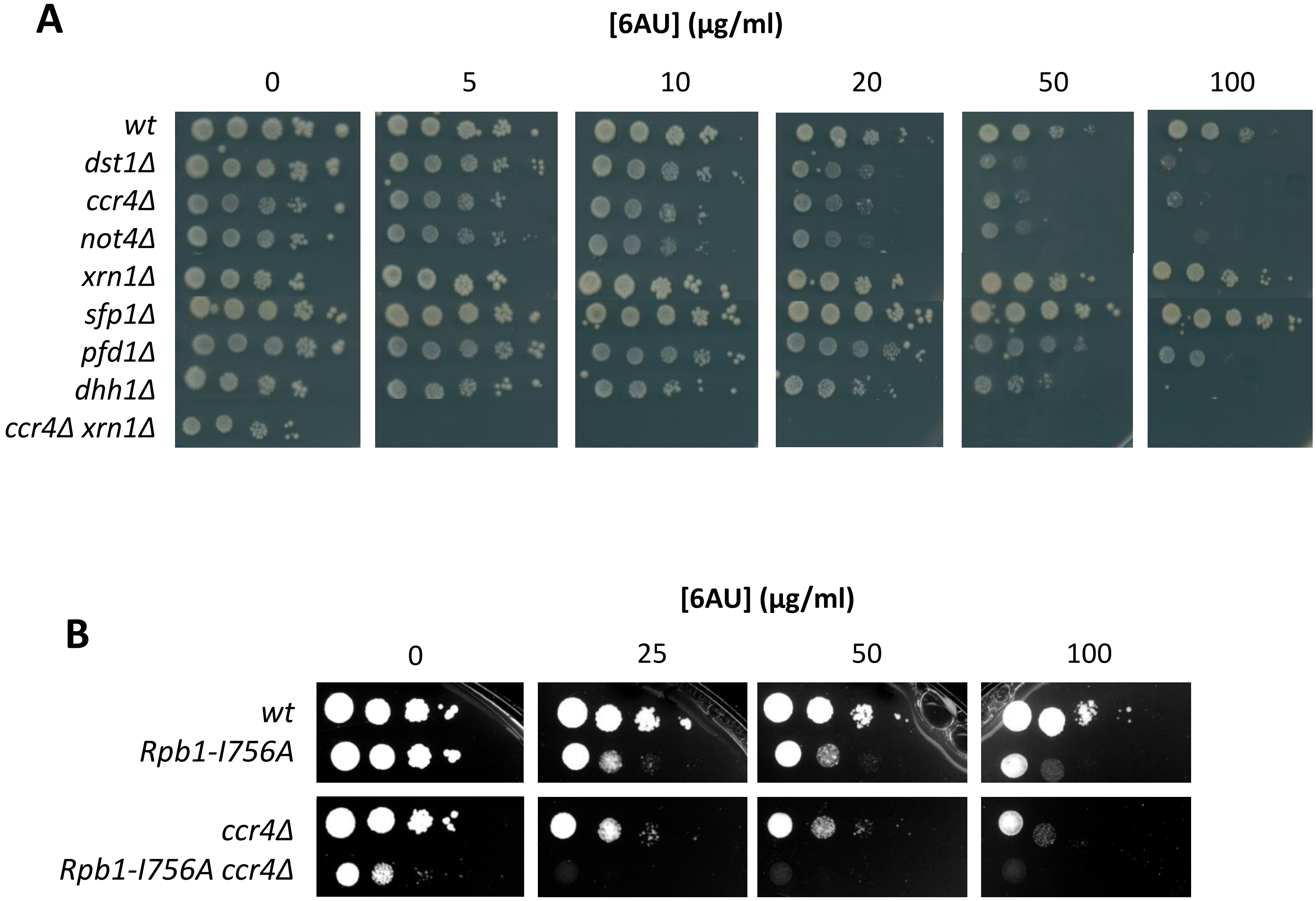

Figure S8. The double *ccr4*Δ *xrn1*Δ mutant is synergistically hypersensitive to 6-azauracil (6AU), an NTP-depleting drug, as compared to the single mutants. A. We show a yeast growth assay, where we spotted decreasing numbers of cells onto SC-Ura plates containing the indicated concentrations of 6AU. *xrn1Δ, sfp1Δ* and *pdf1Δ* all grew as well as the wild type even in 6AU 100 µg/ml. As expected *dst1Δ, ccr4Δ* and *not4Δ* were hypersensitive to 50-100 µg/ml 6AU. *dhh1Δ* showed a vey mild sensitivity, whereas the double *ccr4Δ xrn1Δ* mutant presented a dramatic hypersensitive phenotype, with no growth even at the lowest concentrations of the drug. B. Growth assay of *Rpb1-I567A* and the double mutant *Rpb1-I567A ccr4Δ* in the presence of 6AU. *Rpb1-I567A* is only slightly more sensitive to the drug than the wt, however, in combination with *ccr4Δ* it becomes hypersensitive to 6AU.

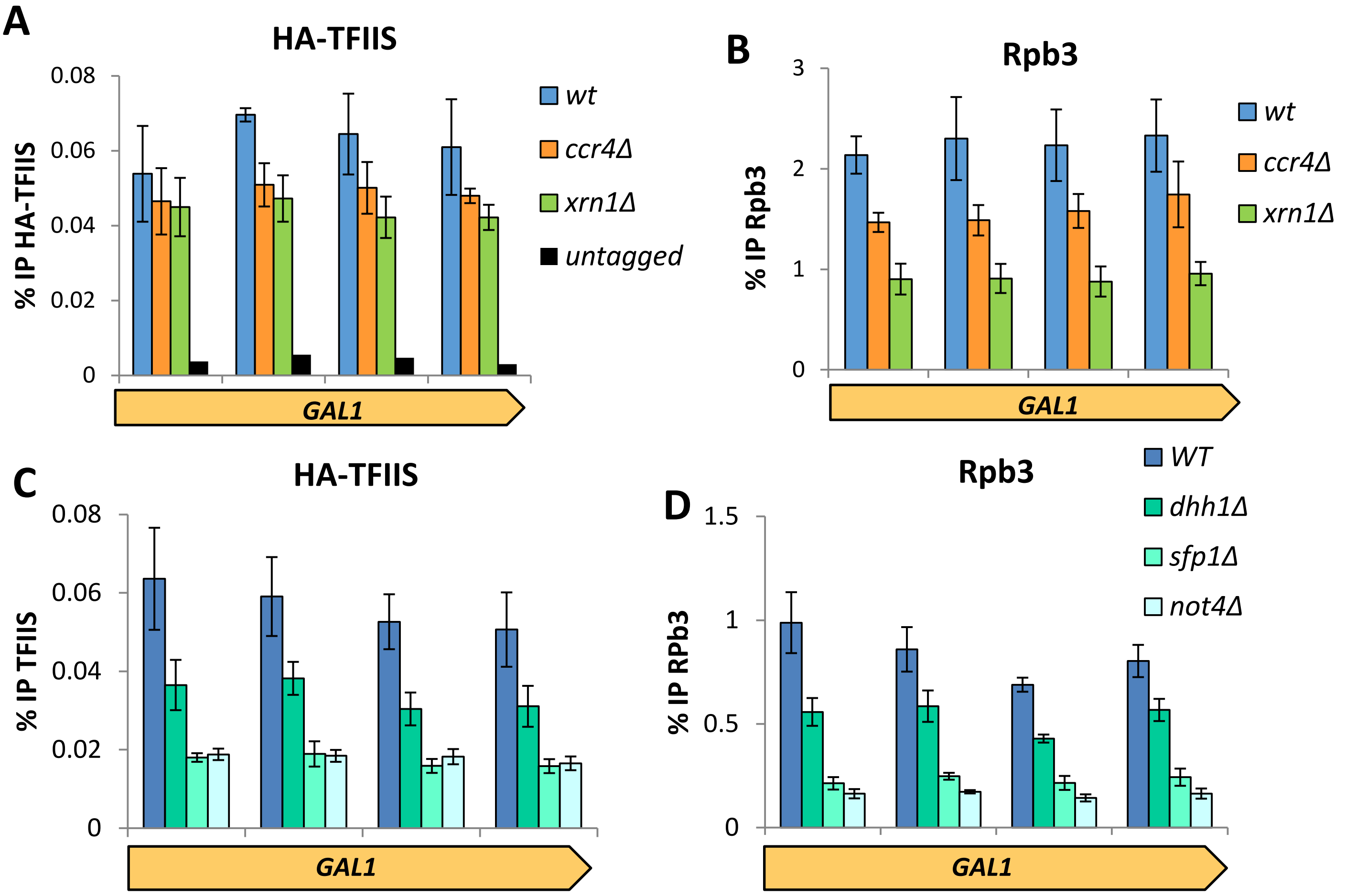

Figure S9. TFIIS and Rpb3 occupancy in *xrn1*Δ, *ccr4*Δ*, dhh1*Δ*, sfp1*Δ *and not4*Δ*.* A. TFIIS occupancy is reduced in both *ccr4Δ* and *xrn1Δ* mutants on the *GAL1* gene. The bars represent ChIP results as % of the input of three biological replicates. B. The occupancy of Rpb3 is also reduced in both mutants compared to the wild type. However, in the case of *xrn1Δ,* Rpb3 levels are decreased more than TFIIS, leading to the increase ratio shown in Fig 5A. C. The occupancy of TFIIS is reduced along the *GAL1* gene in the three additional mutants studied: *dhh1*Δ*, sfp1* Δ and *not4*Δ*.* D. The occupancy of Rpb3 is also lowered in all three mutants, however, the TFIIS/Rpb3 ratio is only significantly higher in *not4*Δ (Fig 5C). The ratio of the other two mutants remains unchanged. Anti-HA and anti-Rpb3 ChIPs were performed with the same extracts in each biological replicate, allowing direct calculation of the TFIIS/Rpb3 ratios shown in Fig 5. An untagged control for the HA-TFIIS ChIP is shown in A.

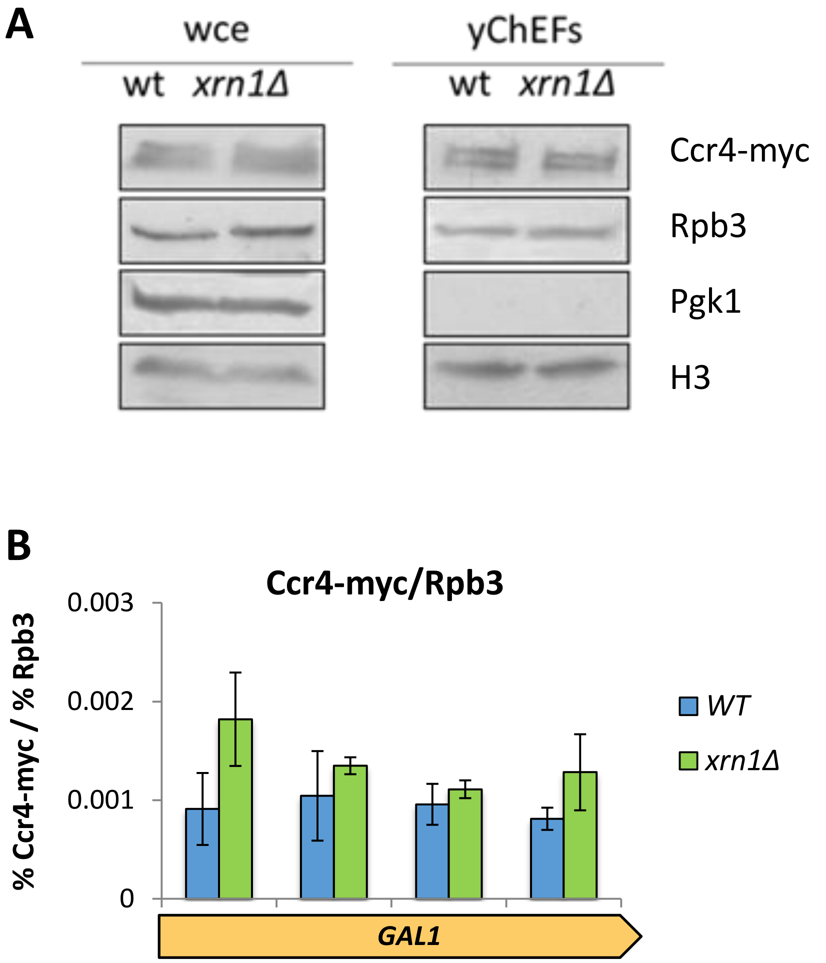

Figure S10. The amount of Ccr4 on genes is unchanged in the *xrn1Δ* strain. A. The amount of Ccr4 on chromatin in an *xrn1Δ* mutant is similar to that in the wild type. We cannot see any significant difference in the amount of Ccr4 in the whole cell extract (wce), nor in the chromatinic fraction (yChef). The total amounts of RNAPII are also monitored using an anti-Rpb3 antibody. Pgk1 is a control that is only present in the cytoplasm (no bands in the yChef fraction); histone H3 is a positive control of the chromatin fraction. B. Results of anti-Myc ChIP experiments, showing the relative occupancy of Ccr4-myc compared to Rpb3. No significant changes can be found when comparing the wild type and *xrn1Δ* mutant strains. The bars represent mean and standard deviation of three biological replicates (* if p<0.05 in a Student's t-test). C.

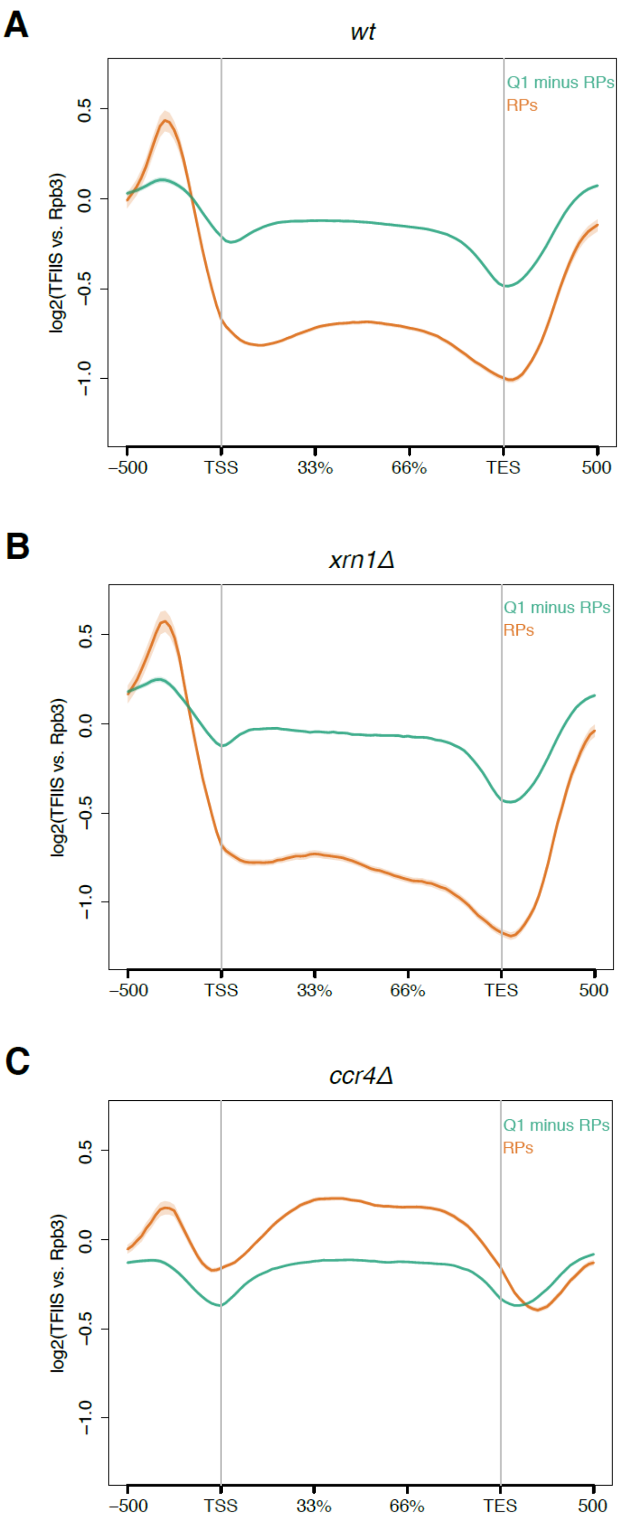

Figure S11. The differential behaviour of the RP gene category in *xrn1*Δ and *ccr4*Δ is not due to its high transcription rate. A. We compare the mean profile of TFIIS/Rpb3 ratios from RP genes (orange) and the genes belonging to the quartile of genes with the highest transcription rates, excluding RP genes (Q1 minus RP, green). TSS is the transcription start site of genes and TES the transcript exit site. The profile is represented as the log2 of the fold change between the TFIIS ChIP and the Rpb3 ChIP. ChIPs of HA-TFIIS and Rpb3 were performed using the same cell extract and in parallel. We represent the mean of two biological replicates. B-C. We have represented the same analysis as in A but for the *xrn1*Δ (B) and *ccr4*Δ mutants (C).

Table S1. The *Saccharomyces cerevisiae* strains used in this work.

Yeast strain	Relevant genotype	Reference
16.53a	*URA3::GAL1p::YLR454w*	Malagón F.
16.53b	*URA3::GAL1p::YLR454w*	Malagón F.
GMY16-8d	*URA3::GAL1p::YLR454w dst1::KanMX4*	Millán-Zambrano et al., 2013
MAY8	*URA3::GAL1p::YLR454w ccr4::KanMX4*	This work
VBY2	*URA3::GAL1p::YLR454w xrn1::KanMX4*	This work
VBY3	*URA3::GAL1p::YLR454w sfp1::KanMX4*	This work
GMY20-5c	*URA3::GAL1p::YLR454w pfd1::kanMX4*	Millán-Zambrano et al., 2013
VBY14	*URA3::GAL1p::YLR454w not4::KanMX4*	This work
VBY50	*URA3::GAL1p::YLR454w dhh1::KanMX4*	This work
VBY56	*URA3::GAL1p::YLR454w rpb1::KanMX4 pFL44L-RPB1::leu*	This work
VBY57	*URA3::GAL1p::YLR454w rpb1::KanMX4 pFL44L-rpb1-I756A::leu*	This work

Table S2. The primers used in this work.

Primer	Sequence 5’-3’	Use
GAL1-1040F	AAGAGTCTCTCGCCAATAAGAAAC	RT-qPCR
GAL1-1142R	GATGTTGTTAAGTAGTCTCTTGTG	RT-qPCR
SCR1_RTup	GTCTCTCTGTCTGGTGCGGC	RT-qPCR
SCR1_RTlow	CACGGGTCACCTTTGCTGA	RT-qPCR
1_100.txt-3F (*YLR454w*)	GATGTTTCCGATTAATGTTCTACTGTACAA	ChIP qPCR
1_100-66R (*YLR454w*)	GCTCCATAAGAAAGTCACTGCAAA	ChIP qPCR
1900-2000-3F (*YLR454w*)	AGACAGAAGGAAATTTTACCAAGCG	ChIP qPCR
1900-2000-63R (*YLR454w*)	AATCGAAAAAATCAGGTAGTTGCTG	ChIP qPCR
3800-4100-191F (*YLR454w*)	GATATGCTTCAATCCGACAGAGAG	ChIP + RT-qPCR
3800-4100-258R (*YLR454w*)	TCAACAGTTACCGATGGTATTAAAGG	ChIP + RT-qPCR
5800-6100-2F (*YLR454w*)	AGCCGGACAAACAGAACAGC	ChIP qPCR
5800-6100-71R (*YLR454w*)	CAGGGTCTTTTTGGTGTTTTTCA	ChIP qPCR
7600_7700.txt-21F (*YLR454w*)	GATTATATGATCGTTGAAGATC	ChIP qPCR
7600_7700.txt-92R (*YLR454w*)	GTTGGACAATCTTAAAGTCGGGA	ChIP qPCR
GAL1-5'(+190) up	CAGAAGAAGTGATTGTACCTGAGTTCA	ChIP qPCR
GAl1-5'(+190) low	TGCTCGGGCACTTTTCG	ChIP qPCR
GAL1-ORF2 2643 up	GGTATGGATCAGGCTGCCTC	ChIP qPCR
GAL1-ORF2 2743 low	AATTGCGGAAATTTAAACGGAG	ChIP qPCR
GAL1-ORF3 1999 up	ATCCGGCATCGAACGGTTA	ChIP qPCR
GAL1-ORF3 2099 low	TCAAGGATTGTGCGACATCG	ChIP qPCR
GAL1-3' 1273 up	CAATTTGGTGCCTTGATGAACGAG	ChIP qPCR
GAL1-3' 1386 low	ACCATATGATCCATTTGACAAAGC	ChIP qPCR

Primer	Sequence 5’-3’	Use
GAL1-5' up	ATGACTAAATCTCATTCAGAA	Run-on Probe
GAL1-5' low	AAATTTGGGATCAGCATTTA	Run-on Probe
GAL1-ORF2 up	TTCTAAAGAAACTTGCACCG	Run-on Probe
GAL1-ORF2 low	GCGGTTTGAACTCAACGTAT	Run-on Probe
GAL1-ORF3 up	CCCAACCAACTATAATTTAA	Run-on Probe
GAL1-ORF3 low	CGAGAACAATTCAAGGATTG	Run-on Probe
GAL1-3' up	GTCTTGAAGGCTGTGAAATT	Run-on Probe
GAL1-3' low	AGGGTACTTGACCTTGTAGA	Run-on Probe

Explanation of half-life

	Speed	Volume	Probability
1	*	*	0.558
2		*	0.435
3			0.005
4	*		0.002

Explanation of volume

	HL	Speed	Probability
1	*		0.573
2	*	*	0.419
3			0.006
4		*	0.001

Table S3. Both RNAPII speed and cell volume independently contributes to mRNA half-life. RNAPII speed correlates with mRNA half-life as well as with cell volume. Cell volume also correlates with mRNA half-life. Therefore we checked through Bayesian statistics if any of these correlations were consequences of the other two. Results showed that the most probable model for explaining mRNA half-life is through both RNAPII speed and volume. In contrast, the most probable model for explaining cell volume is through just mRNA half-life; therefore, the correlation speed-volume is likely a consequence of the correlations speed-half-life and half-life-volume. An asterisk (*) stands for the inclusion of the variable in the model. Bayes factors were calculated using the BayesVarSel package from R-project (*https://cran.r-project.org/web/packages/BayesVarSel/BayesVarSel.pdf*), conceived to calculate Bayes factors in linear models and then to provide a formal Bayesian answer to testing and variable selection problems.

Definition of the models

Definition of the system and its agents.

The most important elements of the mechanism were selected and defined as agents. These agents were:

- A single gene divided into three parts:
    - Promoter: where RNA Polymerase II (Pol II) binds to begin transcription.
    - Body of the gene: the coding part of the gene that contains the information to be transcribed into mRNA.
    - Terminator: part of the gene where the Pol II finishes transcribing and separates from the gene.
- RNA Polymerase II: cellular machinery that is in charge of synthesizing the mRNA.
- mRNA: messenger molecule that codifies a protein that is formed by two regions:
    - Poli(A) tail: the 3' region of the mRNA that will be degraded specifically by Ccr4.
    - Informative region: the codifying part of the mRNA that will be degraded by Xrn1 once the poli(A) tail is degraded.
- Ccr4-Not: the agent that is capable of degrading the poli(A) tail of the mRNA.
- Xrn1: agent that degrades the informative region of the mRNA.
- TFIIS: agent that helps the RNA polymerase II resolve situations of backtrack.

Once the agents were defined, we proceeded to define the virtual world as a single cell. The virtual world is a simulated space where the agents "live" and wander around randomly with a Brownian movement. This space is a cube divided into patches whose spatial position is controlled by whole Cartesian coordinates (x,y,z). Two types of units exist in this world: the nucleus situated in the centre and the cytoplasm. The nucleus is simulated as a smaller cube with the gene in the centre and permeable walls that permit the entrance and exit of the agents. In the nucleus we can usually find the gene, Pol II, TFIIS and mRNA agents; whereas in the cytoplasm we can typically find the mRNA, Ccr4-Not and Xrn1 agents.

The agents posses different individual states:

- Promoter:
    1. Open state
    2. Closed state
- RNA Polymerase II:
    1. Random Brownian movement.

2. Moving along the gene engaged in mRNA synthesis in a forward motion.
3. Backtracking state where it randomly makes a backward motion and becomes paused.

- mRNA:
  1. Random Brownian movement.
  2. Bound to Ccr4-Not who is degrading its poly(A) tail.
  3. Searching for Xrn1 once the poly(A) tail has been degraded.
  4. Bound to Xrn1 who completes its total degradation.
- Ccr4-Not:
  1. State where it has not degraded the poly(A) tail of the mRNA.
  2. Active state where it has degraded the poly(A) tail and can enter the nucleus and act upon active or backtracked Pol II.
  3. State where it can bind the nascent mRNA and aid in its export and degradation.
- Xrn1:
  1. State where it has not degraded the rest of the mRNA.
  2. Active state once it has degraded the mRNA who can enter the nucleus and act upon the gene promoter.

The behaviour of each agent was defined using flow charts where the principal function, the interactions with other agents and the repercussions of those actions were established (figures 1-7).

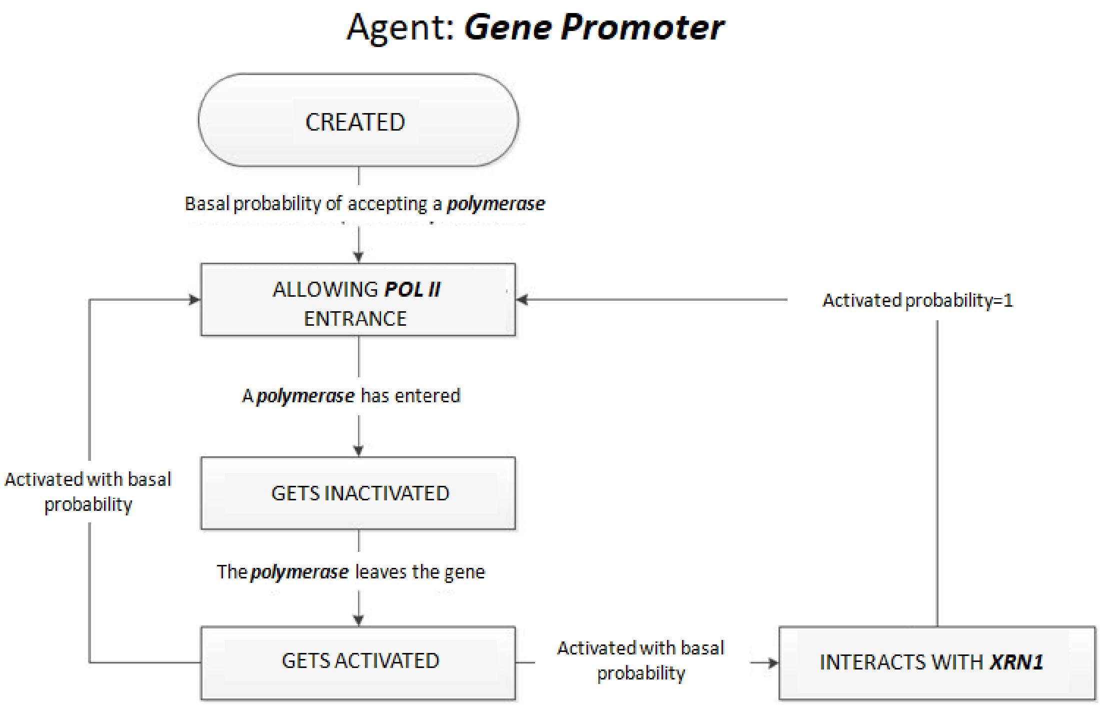

Figure 1. Flow chart for gene promoter. In this flow chart the behaviour of the gene promoter is defined as well as it's interaction with other agents.

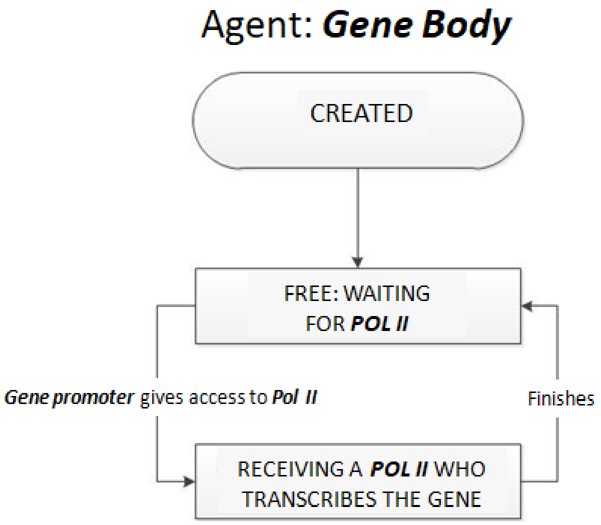

Figure 2. Flow chart for the gene body.

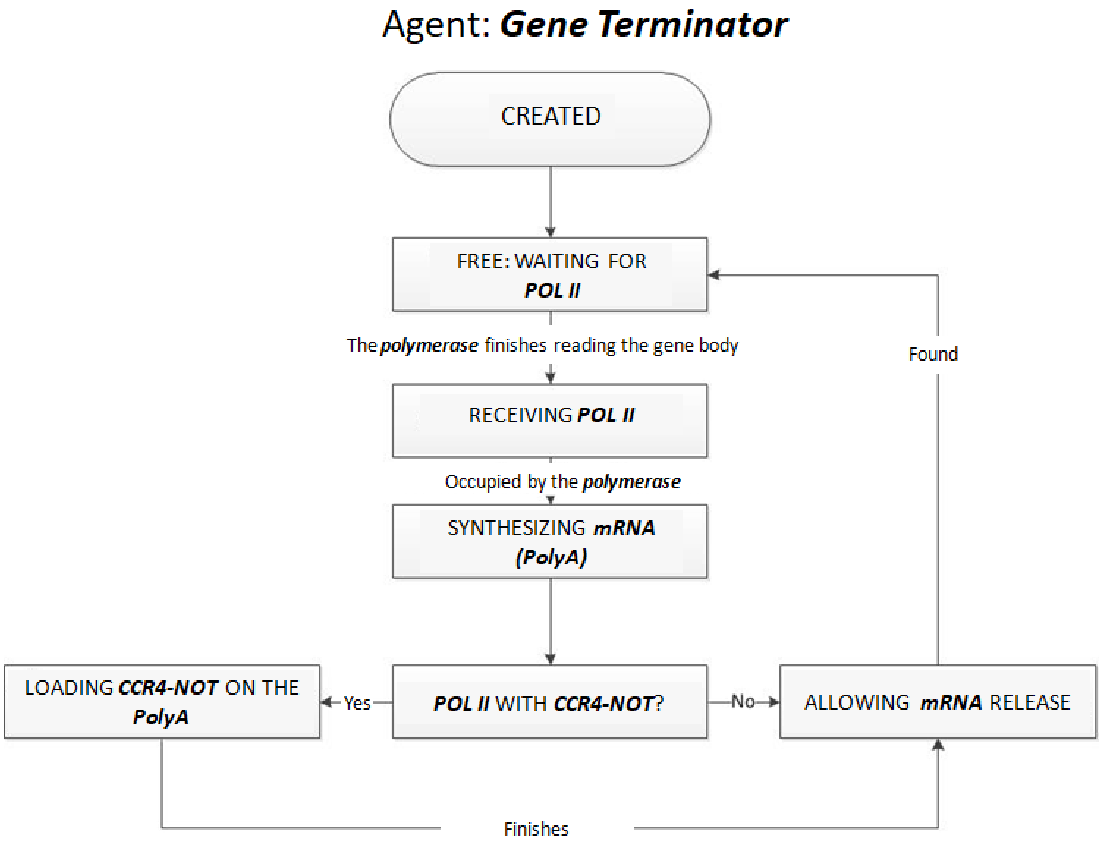

Figure 3. Flow chart for the gene terminator.

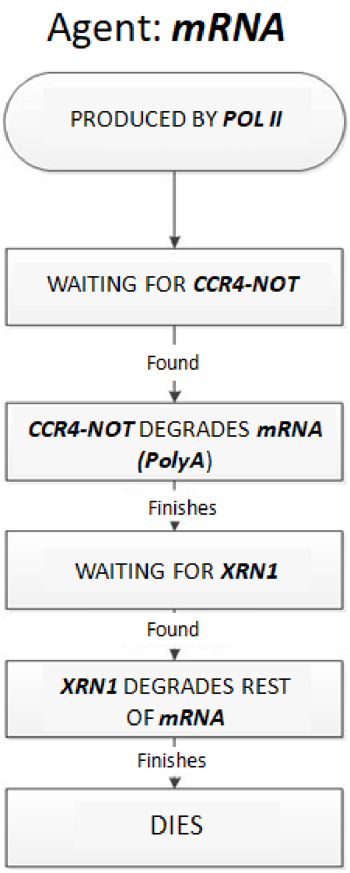

Figure 4. Flow chart for the messenger RNA (mRNA).

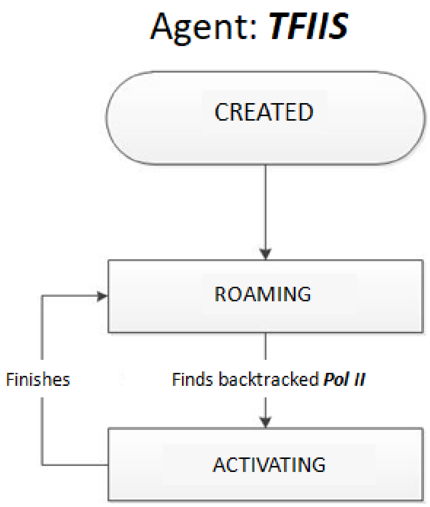

Figure 5. Flow chart for TFIIS.

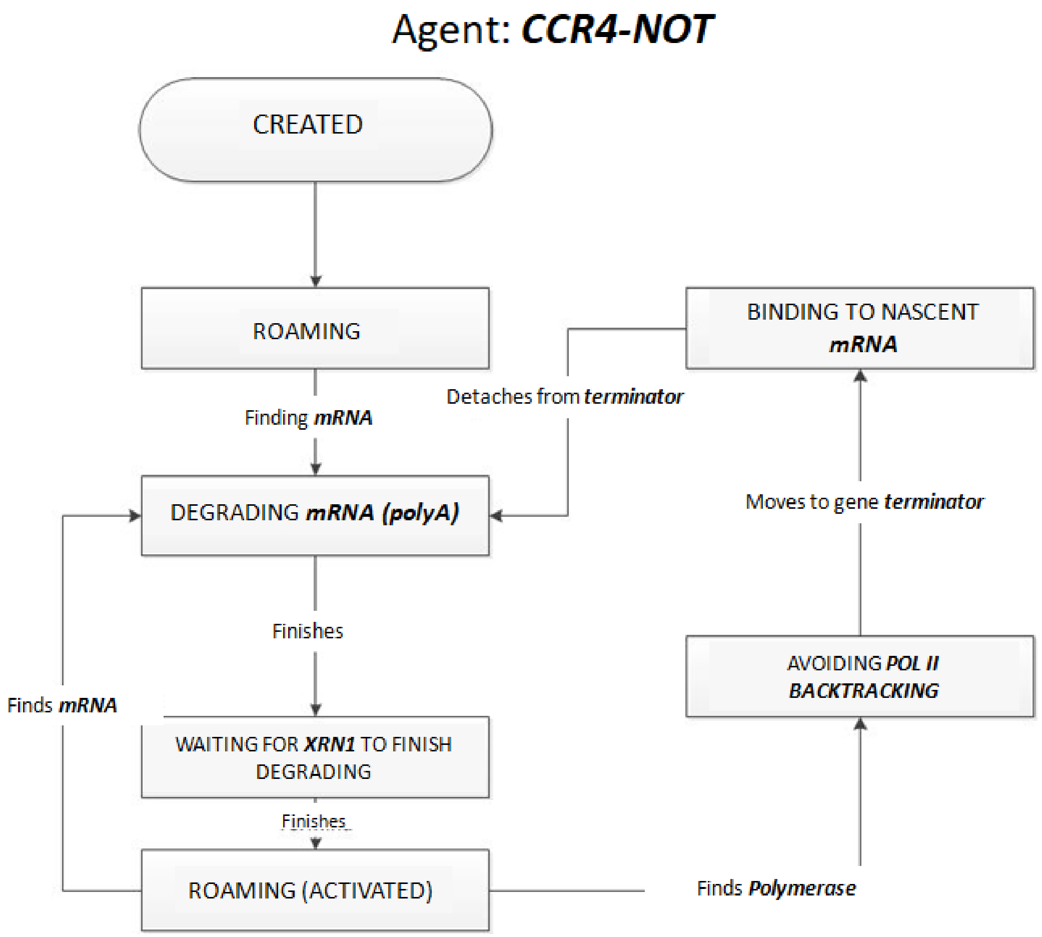

Figure 6. Flow chart for Ccr4-Not.

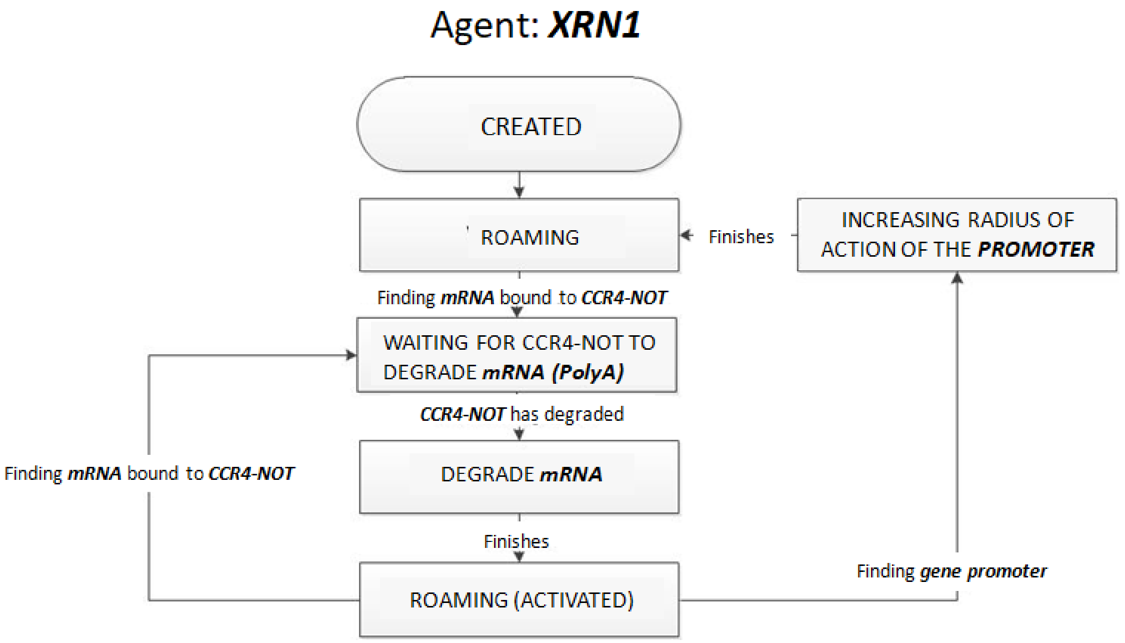

Figure 7. Flow chart for Xrn1.

To simulate the evolution of the agents and their interactions in the virtual world we needed to establish the passage of time. The temporal evolution of the system is divided into equal intervals associated to steps in the program which we call “tick”. We arbitrarily set 10 ticks to correspond to 1 second of real time. The total duration of the simulation can be changeable depending on the objectives we wanted to accomplish.

If all the agents are defined and characterized adequately, we expect an emergent property in the system. This is a property that we have not intentionally defined but appears as a consequence of the interaction of the different parts of the system. In our case, we expect the emergent property to be a steady state level of mRNA that remains constant over time thanks to a balance between mRNA synthesis and degradation.

Programming the system.

The simulation was developed using NetLogo. The first versions of the model was programmed in a 2D using the NetLogo version 5.2.1 platform. Nevertheless, shortly afterwards it was changed to the 3D using the NetLogo version 6.0.2.

The simulation starts with randomly distributed agents in the virtual world, except for the gene which is always in the centre of the nucleus. Then the agents follow the behaviour rules previously defined on a loop until it reaches the final number of steps established in the program. To achieve the proposed goal, we needed to be able to compare experimental data generated in the laboratory with data obtained computationally with the agent-based simulation model. Therefore, we monitored certain variables throughout the execution of the program that are equivalent to the in vivo experimental data. The variables monitored were: total amount of mRNA, mRNA half-life, the proportion of time that the gene is occupied by Pol II (total Pol II), and the proportion of time that the gene is occupied by an active Pol II (Pol II activity). The program shows a graphic view of the number of mRNAs over time and it gives a final report with the numeric values for the controlled variables (see videos).

Once we had defined the agents and behaviours in the first simulation, we adjusted each of the initial parameters of the system to real values that were found in scientific papers (table 1). We decided to model the *GAL1* gene of *Saccharomyces cerevisiae,* which is an inducible gene of high expression that can be easily studied in the laboratory.

Parameter	***Fixed value***	***Reference***
Gene size	1587 pb	Saccharomyces Genome Database
mRNA size	1587 + 70 pb PoliA tail	Brown and Sachs, 1998
Number of Pol II	6	Pelechano et al., 2010
Number of TFIIS	6	Pelechano et al., 2010
Number of Ccr4-Not	36	Pelechano et al., 2010
Number of Xrn1	116	Pelechano et al., 2010
Transcription rate	2 Kb/min	Mason and Struhl, 2005
Poly (A) degradation rate by Ccr4-Not	13 nt/min	Tucker et al., 2001
Poly (A) degradation rate in *ccr4Δ*	2.5 nt/min	Tucker et al., 2001
5' mRNA degradation rate by Xrn1	250 nt/min	Haimovich et al., 2013a
5' mRNA degradation rate in *xrn1Δ*	63 nt/min	Haimovich et al., 2013a
Backtracking probability	95%	Probability of Pol II backtracking
Probability Open	1	Probability of finding an open promoter to start transcription.
Probability React Pol II	2	Probability of reaction from Pol II.
Probability React Help Pol II	50	Probability of reaction from Pol II in the model variant Help Pol II.
Radio Action	2	Radius of action for most agents.
Radio Action Ccr4-Not	5	Radius of action specific to Ccr4-Not.
Radio Action TFIIS	1	Radius of action specific to TFIIS.
Radio Action TFIIS - Help TFIIS	3	Radius of action specific to TFIIS in the help-TFIIS model variant.
Radio Action Promoter	5	Radius of action specific to the promoter. It is increased when Xrn1 acts.

Table 1. Parameters used in model 8.3 (the first model). In the *Saccharomyces Genome Database* we found the total number of molecules in a complete cell. We deducted the molecules corresponding to a single *GAL1* gene by using its transcription rate (0,1%) and percentage of mRNA (1,15%) with respect to the total amount in a cell (Pelechano et al., 2010). The degradation rate by Xrn1 was deducted from *GAL1* mRNA half-life (Haimovich et al., 2013a) and Ccr4-Not degradation rate (Tucker et al., 2001). With this data, the time in which Ccr4-Not degrades the poly(A) tail was calculated and we assumed the rest of the time taken to completely degrade the mRNA was the amount of time it took Xrn1 to degrade the rest. The rates found in the literature in nt/min were transformed to nt/tick.

Once the parameters were fixed, we executed the program to see how it behaved. If everything was working correctly, we expected to obtain the emergent property, which was a stable stationary level of mRNA. This result was quickly achieved (see Suppl figure S5 and video).

Based on experimental results and the results from the first model, we decided to create a second model that reflected our findings. There is a scheme of how the second model works in figure 3D. We added some new parameters for this new model and discarded some of the previous model. The new parameters can be found in table 2.

Parameter	***Fixed value***	***Reference***
Gene size	1587 pb	Saccharomyces Genome Database
mRNA size	1587 + 70 pb PoliA tail	Brown and Sachs, 1998
Number of Pol II	6	Pelechano et al., 2010
Number of TFIIS	8	Pelechano et al., 2010
Number of Ccr4-Not	12	Pelechano et al., 2010
Number of Xrn1	24	Pelechano et al., 2010
Transcription rate	2 Kb/min	Mason and Struhl, 2005
Poly (A) degradation rate by Ccr4-Not	13 nt/min	Tucker et al., 2001
Poly (A) degradation rate in *ccr4Δ*	2.5 nt/min	Tucker et al., 2001
5’ mRNA degradation rate by Xrn1	250 nt/min	Haimovich et al., 2013a
5’ mRNA degradation rate in *xrn1Δ*	63 nt/min	Haimovich et al., 2013a
Backtracking probability	5%	Darzacq et al., 2007
Probability Open	1.268%	Probability of finding an open promoter to start transcription.
Probability React Pol II	16%	Probability of reaction from Pol II.
Radio Action	2	Radius of action for most agents.
Radio Action Ccr4-Not	3	Radius of action specific to Ccr4-Not.
Radio Action TFIIS	1	Radius of action specific to TFIIS.
Radio Action Promoter	3	Radius of action specific to the promoter. It is increased when Xrn1 acts.
Probability State I	10	Probability of Pol II of falling into an inactive state

Drop-off threshold	714	Probability of Pol II dropping off the gene after becoming backtracked if not resolved by TFIIS
IS x BTS	5	Time in ticks for which Pol II becomes invulnerable to becoming inactive when Xrn1 acts

Table 2. Parameters used in model 10.1 (the second model). We eliminated some of the parameters used in the previous model and added others to satisfy our new model needs.
